# Trimodal distribution of arylamine N-acetyltransferase 1 mRNA in breast cancer tumors: association with overall survival and drug resistance

**DOI:** 10.1186/s12864-018-4894-4

**Published:** 2018-07-03

**Authors:** Rodney F. Minchin, Neville J. Butcher

**Affiliations:** Laboratory for Molecular and Cellular Pharmacology, School of Biomedical Sciences, University of Queensland, Brisbane, QLD Australia

**Keywords:** Breast cancer, Arylamine N-acetyltransferase, Drug resistance, Overall survival

## Abstract

**Background:**

Arylamine N-acetyltransferase 1 (NAT1) is a drug metabolizing enzyme that has been associated with cancer cell proliferation in vitro and with survival in vivo. NAT1 expression has been associated with the estrogen receptor and it has been proposed as a prognostic marker for estrogen receptor positive cancers. However, little is known about the distribution of NAT1 mRNA across an entire patient population or its effects on outcomes. To address this, gene expression data from breast cancer patient cohorts were investigated to identify sub-populations based on the level of NAT1 expression. Patient survival and drug response was examined to determine whether NAT1 mRNA levels influenced any of these parameters.

**Results:**

NAT1 expression showed a trimodal distribution in breast cancer samples (*n* = 1980) but not in tumor tissue from ovarian, prostate, cervical or colorectal cancers. In breast cancer, NAT1 mRNA in each sub-population correlated with a separate set of genes suggesting different mechanisms of NAT1 gene regulation. Kaplan-Meier plots showed significantly better survival in patients with highest NAT1 mRNA compared to those with intermediate or low expression. While NAT1 expression was elevated in estrogen receptor-positive patients, it did not appear to be dependent on estrogen receptor expression. Overall survival was analyzed in patients receiving no treatment, hormone therapy or chemotherapy. NAT1 expression correlated strongly with survival in the first 5 years in those patients receiving chemotherapy but did not influence survival in the other two groups. This suggests that low NAT1 expression is associated with chemo-resistance. The sensitivity of NAT1 mRNA levels as a single parameter to identify non-responders to chemotherapy was 0.58 at a log(2) < 6.5.

**Conclusions:**

NAT1 mRNA can be used to segregate breast cancer patients into sub-populations that demonstrate different overall survival. Moreover, low NAT1 expression shows a distinct poor response to chemotherapy. Analysis of NAT1 expression may be useful for identifying specific individuals who would benefit from alternative therapy or drug combinations. However, additional information is required to increase the sensitivity of identifying non-responders.

**Electronic supplementary material:**

The online version of this article (10.1186/s12864-018-4894-4) contains supplementary material, which is available to authorized users.

## Background

Breast tumors arise primarily from the epithelial cells in the milk ducts. However, they exhibit considerable histological heterogeneity not only between patients but also within patients. In the past, histological grading of breast cancers, hormone receptor status and lymph node involvement have been used to help guide treatment, with better survival outcomes in many patients [1]. More recently, molecular classification by gene expression has added significantly to our understanding of breast cancer heterogeneity and has provided additional information for developing treatment strategies [2]. Nevertheless, there remains significant sub-populations that do not respond well to therapy based on their histopathological and/or molecular characterization.

Arylamine N-acetyltransferase 1 (NAT1) is one of two human enzymes that metabolizes arylamine and hydrazine-type drugs [3]. The gene that encodes NAT1 resides on chromosome 8 and is genetically polymorphic [3]. Apart from its role in biotransformation, NAT1 has also been associated with cancer cell growth and invasion [4–6]. Thus, it joins a number of other drug metabolising enzymes such as Cyp2E1 [7], glutathione transferases [8] and UDP glucuronyltransferases [9] that have been shown to affect cell proliferation.

The expression of NAT1 in over 40 cancer microarray studies was recently reviewed and a number of cancers showed significant differences between normal and malignant tissues [3]. The most striking examples were seen in breast cancer array data where NAT1 up-regulation was commonly associated with estrogen receptor (ER) positive tumors [10–12]. Indeed, NAT1 has been proposed as a prognostic marker for ER positive breast cancer [13]. NAT1 is included in the Prosigna Breast Cancer Prognostic Gene Signature Assay (PAM50) [14], which has proven to be useful in identifying patients most likely to benefit from drug treatment [15]. NAT1 mRNA is also elevated in male breast cancer [16, 17] and breast tumors that preferentially metastasize to the bone [18, 19]. Interestingly, it is significantly down-regulated in early onset breast cancer [20].

Why NAT1 might influence cell proliferation in vitro or invasion in vivo is unknown. Moreover, its role in breast cancer patient survival has not been determined. Along with the estrogen receptor 1 gene (ESR1), NAT1 segregates with GATA3 and FOXA1 in ER-positive tumors [21]. Both GATA3 and FOXA1 form complexes with ESR1 to initiate expression of estrogen-responsive genes [22]. It is possible that the up-regulation of NAT1 is a response to these transcriptional factors. However, while NAT1 expression is increased by androgens [23], is does not appear to be regulated by ER or the ER/FOXA1/GATA3 complex [22]. Nor is it responsive to estrogen in breast cancer cells [24]. NAT1 expression may simply be another prognostic marker for ER positive tumors. Alternatively, it may have a biological role in cell growth and survival in vivo such that understanding of its expression could lead to alternative approaches to treatment. The current study was undertaken to address these different possibilities and to determine whether NAT1 influences patient survival. To do this, curated gene expression data from extensively described breast cancer patient cohorts were investigated to identify sub-populations based on the level of NAT1 expression. Other genes that segregated into these sub-populations were also identified. Finally, patient survival and drug response was examined to determine whether NAT1 mRNA levels influenced any of these parameters.

## Results

### NAT1 expression in breast cancer patients shows multiple sub-populations

**Fig. 1:**
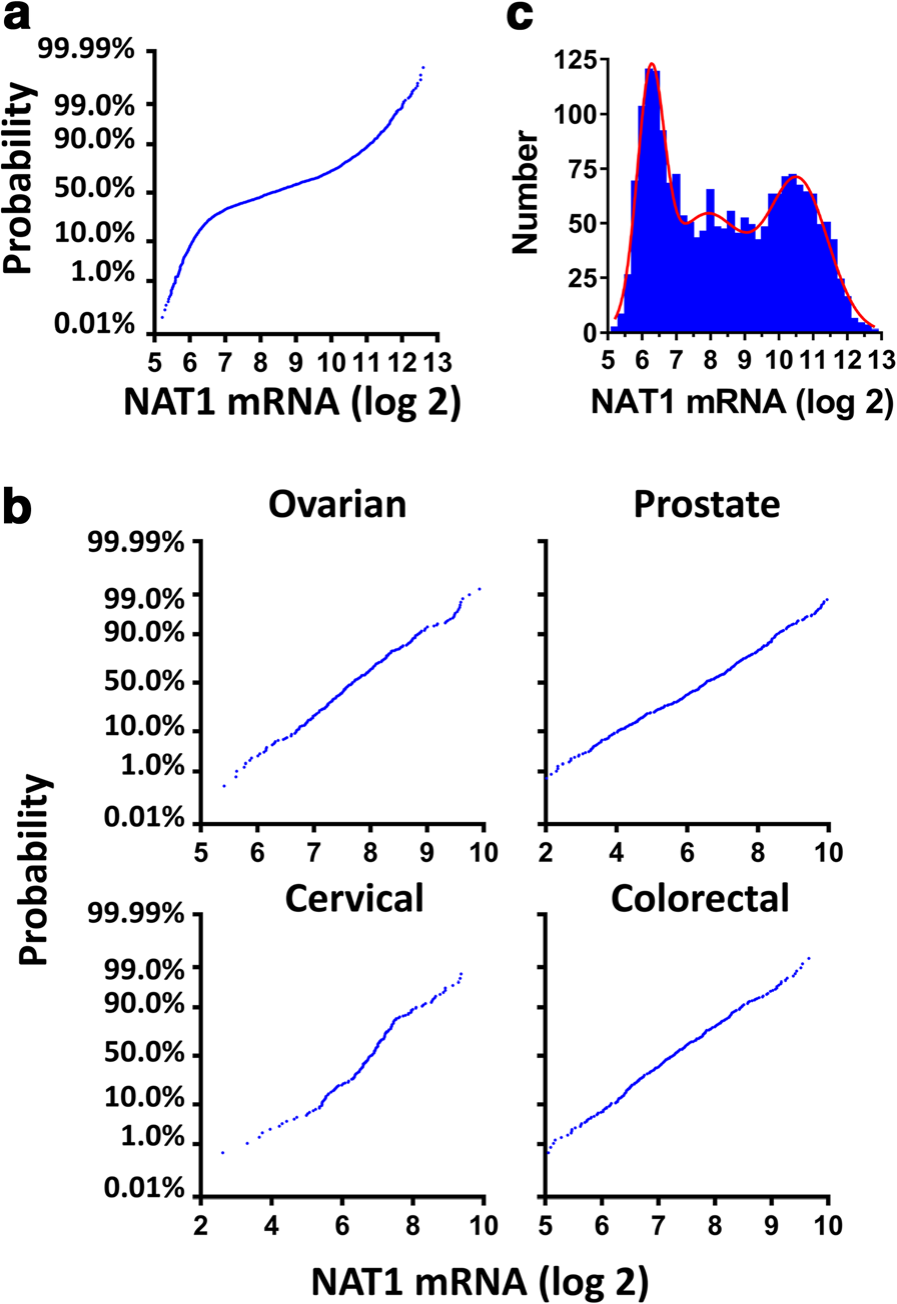
Distribution of NAT1 mRNA in cancer patients. **a** Probit plot of NAT1 mRNA for 1980 patient samples. Deviation from linearity was confirmed by the Kolmogorov-Smirnov Normality Test (K-S distance = 0.0574, *p* < 0.0001). **b** Probability plots for NAT1 mRNA in ovarian (*n* = 299), prostate (*n* = 491), cervical (*n* = 190) and colorectal (*n* = 383) cancer patient samples (from TCGA). **c** Frequency plot of NAT1 mRNA in breast cancer patients. Multiple Gaussian distribution fitted by nonlinear regression is shown in red

NAT1 expression in breast cancer was examined by Probit analysis using data from METABRIC (*n* = 1980). Fig. 1a shows NAT1 mRNA levels (log(2) transformed) in these patients. The distribution was not normal, as demonstrated by the non-linear Probit plot (Kolmogorov-Smirnov Normality Test, K-S distance = 0.0574, *p* < 0.0001). By contrast, NAT1 expression in ovarian, prostate, cervical and colorectal cancers showed single, normally-distributed populations (Fig. 1b). The non-normal distribution of NAT1 transcripts in breast cancer was not unique to patients in the METABRIC cohort as a similar distribution was seen in patients from the Cancer Genome Atlas database (*n* = 1100) and in patients from Ciriello et al. (*n* = 814) [25] (Additional file 1: Figure S1).

To identify the number of specific NAT1 sub-populations, the frequency data were numerically fitted to multiple Gaussian distributions. The results showed the presence of three distinct sub-populations of NAT1 mRNA – low, intermediate and high expression (Fig. 1c, red line). The parameters for each of these distributions are shown in Table 1. The mean log2 mRNA levels were 6.25, 7.89 and 10.58, respectively, which represents a 20-fold difference between the low and high groups. The number of patients in each sub-population was estimated from the area under the Gaussian curves. There were 515 patients (26%) who expressed low NAT1, 752 (38%) who expressed intermediate NAT1 and 713 (36%) with high NAT1. When a similar multiple Gaussian distribution was fitted to the data shown in Additional file 1: Figure S1, a trimodal distribution best described NAT1 mRNA expression. These results show that NAT1 expression is heterogeneous in breast tumors with at least 3 different mechanisms that regulate its mRNA levels.

**Table 1:** Gaussian distributions for NAT1 mRNA populations in breast cancer

	Low NAT1 Gaussian distribution				Intermediate NAT1 Gaussian distribution				High NAT1 Gaussian distribution			
	Amp	Mean	SD	Patients (%)	Amp	Mean	SD	Patients (%)	Amp	Mean	SD	Patients (%)
ALL	104	6.25	0.39	26%	54	7.89	1.12	38%	68	10.58	0.86	36%
ER+	35	6.47	0.37	10%	49	8.07	1.10	44%	67	10.61	0.84	46%

*Amp* Amplitude, *SD* Standard deviation of the mean, Patients in each population were estimated from the area under the Gaussian distributions as a percent of total area

### Differential gene expression in NAT1 sub-populations

**Fig. 2:**
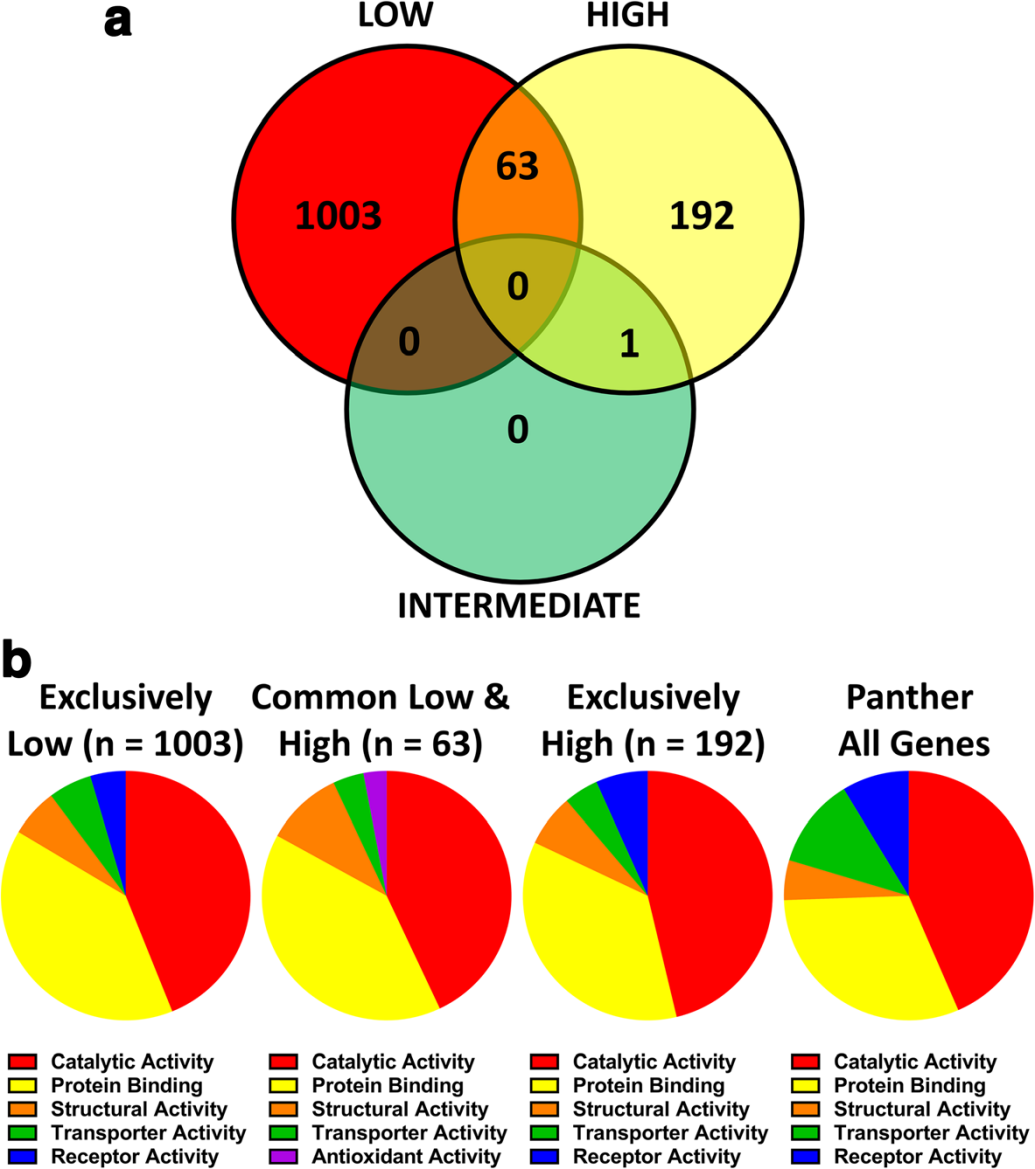
**a** Three-way Venn diagram showing the number of genes with expression that correlated with that for NAT1 using a Pearson’s correlation coefficient with probability adjusted for multiple comparisons. **b** Distribution of molecular functions for genes in the Low and High NAT1 populations as well as those common to both groups. The overall distribution of each function in the Panther database is also shown

The 3 different sub-populations of NAT1 suggest different mechanisms that regulate NAT1 mRNA. One approach to investigate this possibility is to compare co-expressed genes in each of the sub-populations.To do this, patients were divided into low, intermediate and high NAT1 expression with log2 cut-off boundaries from 5 to 6.5, 7.25–8.5 and 10–13, respectively (see Methods). Using Pearson’s correlation coefficient with significance adjusted for multiple comparisons, genes that co-expressed with NAT1 in each of these groups were identified and sorted using a Venn diagram (Fig. 2). While there were 1066 and 255 genes that co-expressed with NAT1 in the low and high groups, respectively, there was only 1 in the intermediate group. The specific genes in each group are listed in Additional file 2: Table S1. The low overall number of genes common between groups supports the notion that different transcriptional/post-transcriptional mechanisms regulate NAT1 expression in the different sub-populations.

Each of the genes that correlated with NAT1 expression in the low and high sub-populations was categorized according to its molecular function using the Panther software [26]. There was a surprising similarity between the two groups with more than 80% of the genes categorized as “catalytic activity” or “protein binding” (Fig. 2b). Moreover, those genes that were common to the two sub-populations also showed a similar functional distribution. When compared to the percent of all genes in each category (Fig. 2b, right graph), genes that co-expressed with NAT1 were similarly distributed suggesting no specific pathway unique to each group. In support of this, an analysis of molecular functions over-represented in the two NAT1 sub-populations showed none in the high NAT1 group and only 1 in the low NAT1 group, which was “translation regulation” with a 3.56-fold enrichment (*p* = 0.0019).

### NAT1, clinical attributes and estrogen receptor expression

We analysed the relationship between NAT1 mRNA expression and various clinical attributes. When the patients were divided according to their PAM50 classification, NAT1 expression was significantly higher in the Luminal A and B groups compared to basal and HER2 positive groups (Additional file 3: Figure S2A). By contrast NAT1 mRNA levels were not associated with Nottingham’s prognostic index, histological grade or tumor stage (Additional file 3: Figure S2 B-D). There was a significant correlation between age at diagnosis and NAT1 expression (*r* = 0.175, *p* < 0.0001). However, this relationship explained less than 4% of the variation between the 2 variables, suggesting very little physiological relevance (Additional file 3: Figure S2E). A similar significant correlation was seen between NAT1 mRNA and tumor size, with an equally weak relationship (*r* = − 0.07, *p* = 0.0016).

**Fig. 3:**
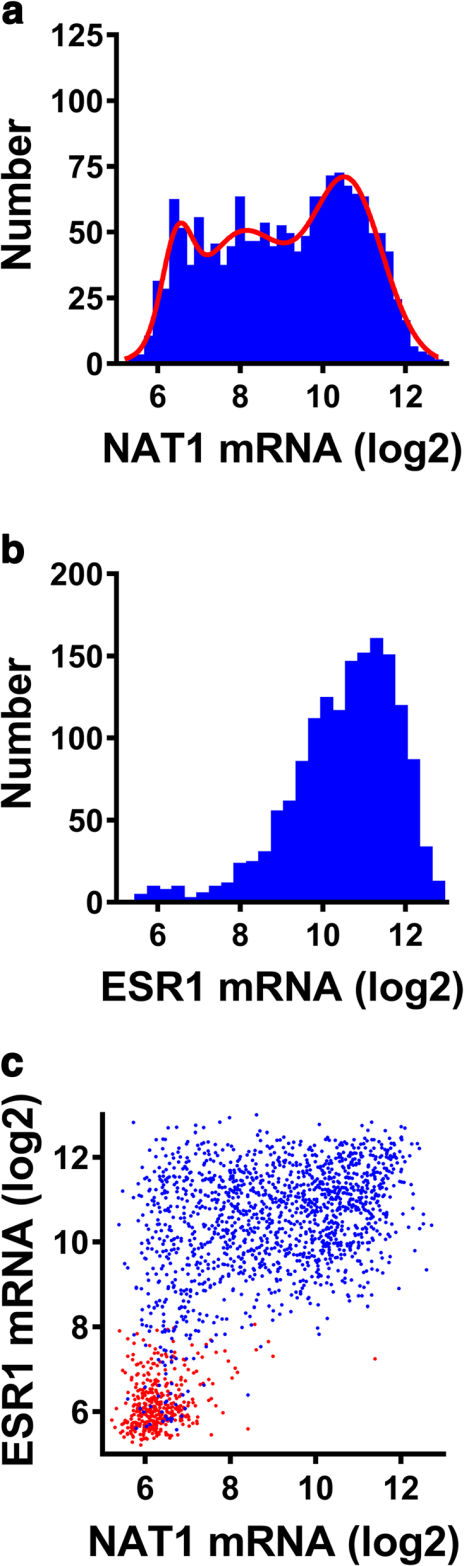
NAT1 and estrogen receptor (ESR1) expression. **a** Frequency distribution of NAT1 mRNA levels in ER positive patients (*n* = 1546). **b** Frequency distribution of ESR1 mRNA levels in ER positive patients (*n* = 1546). **c** Relationship between NAT1 mRNA and ESR1 mRNA in all patients. Red symbols represent those patients diagnosed as ER negative

The higher levels of NAT1 in luminal versus basal breast cancer suggests a relationship with ER expression. NAT1 has previously been qualitatively associated with ER both in microarray studies as well as histological and cell studies [13, 27–29]. NAT1 mRNA levels in ER positive patients, based on their clinical diagnoses, were analyzed using a frequency plot (Fig. 3). The ER positive patients showed a trimodal Gaussian distribution of NAT1 mRNA similar to the entire population (Fig. 3a). However, when the frequency distribution of transcripts for the ER gene (ESR1) was examined, a trimodal distribution was not observed (Fig. 3b). Although the correlation between NAT1 and ESR1 expression for all patients was 0.60 (Spearman’s correlation coefficient), the relationship showed two distinct populations (Fig. 3c). One population consisted of patients with low ESR1 and low NAT1 expression (diagnosed as ER negative - red symbols). The second consisted of high ESR1 (log(2) mRNA > 8) but NAT1 ranging from low to high expression (blue symbols). When the ER positive patients were analyzed separately, Spearman’s correlation coefficient for ESR1 and NAT1 expression decreased to 0.3 indicating that, in this group, ESR1 expression explained less than 10% of the variation in NAT1 expression. These results suggest that much of the association between these two genes reported previously is due to the low, or negligible, expression of NAT1 in patients with low ESR1 expression.

The possibility that patients with low NAT1 were simply non-expressers of the gene (background response on the microarray) was assessed by examining the frequency distribution of the Y chromosome gene SRY on the assumption that this gene was absent in the patient cohort. The SRY mRNA frequency distribution showed a mean value of 5.4 (Additional file 4: Figure S3), which is well below that for NAT1 expression in any of the different populations.

### NAT1 sub-populations and breast cancer survival

**Fig. 4:**
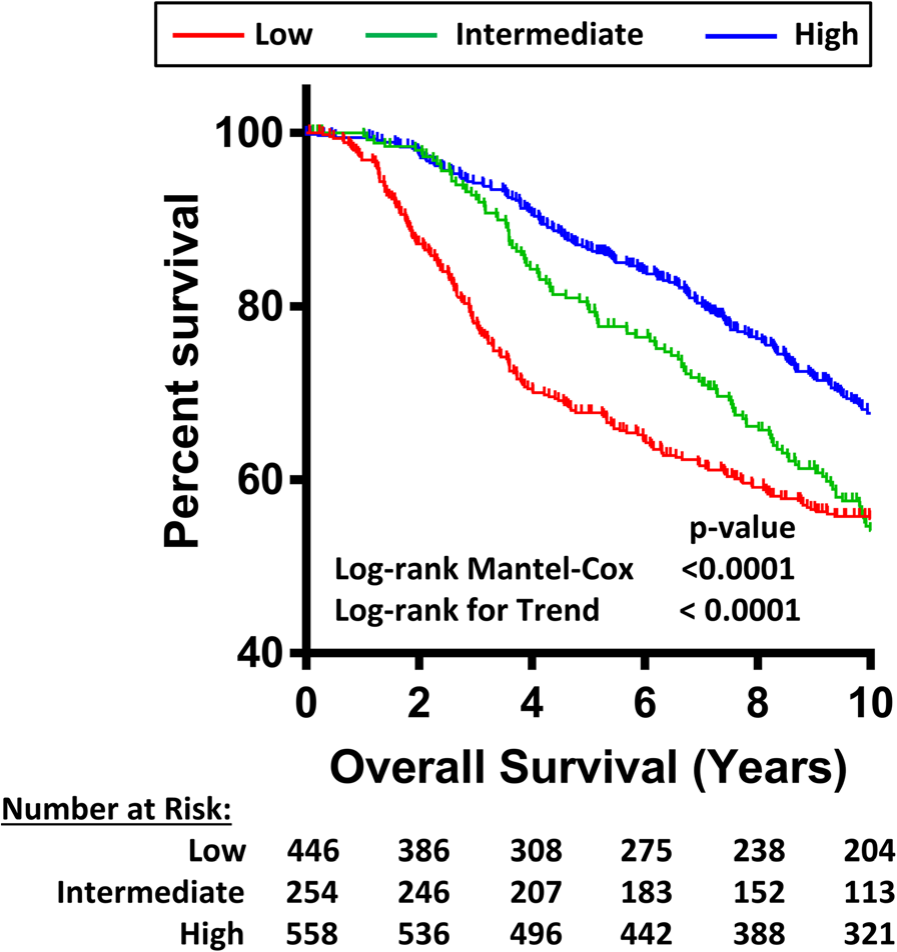
Kaplan-Meier plot of patients in the low, intermediate and high NAT1 sub-populations. Number of patients at risk at 2 year intervals is also shown

The overall survival of patients in each of the NAT1 sub-populations was analyzed using Kaplan-Meier plots (Fig. 4). Over 10 years, there was a significantly better survival in patients with high NAT1 compared to those with low NAT1 (Log-rank Mantel-Cox test *p* = 1.9 E-7), while the intermediate population plotted between the high and low NAT1 groups (Log-Rank test for trend *p* = 1.1E-7). By year 10, 46% of the low NAT1 patients remained at risk while 44% of the intermediate group remained at risk. Thus, the overall survival was similar at 10 years. The major difference was a more rapid decline in survival from year 1 to 4 in the low NAT1 cohort, which was confirmed by the highly significant Gehan-Breslow-Wilcoxon statistic for these 2 groups (*p* = 0.021), which places more weight on events that occur early in overall survival. In patients with high NAT1 expression, 58% remained at risk after 10 years. These data show that the increase in NAT1 expression in the different sub-populations is associated with a significant better overall survival.

To confirm this association, Kaplan-Meier plots were generated for NAT1 mRNA using independent data from Gyorffy et al. [30], which comprised 1402 breast cancer samples (http://kmplot.com/analysis/). These samples were divided into high and low NAT1 around the median mRNA value. Survival was significantly greater (*p* = 1.2E-7) in the high NAT1 group (Additional file 4: Figure S4). This result confirms the survival advantage of elevated NAT1 expression in breast cancer.

Finally, the association between survival and NAT1 sub-populations, tumor size, age at diagnosis, and treatment was investigated using univariate and multivariate Cox proportional hazard regression models (Additional file 5: Table S2). Increasing NAT1 mRNA significantly decreased the hazard ratio (HR) consistent with the results in Fig. 4. By contrast, both increasing tumour size and age at diagnosis increased HR. Neither menopausal state nor radiotherapy or hormone therapy were significant variables in either model whereas chemotherapy increased HR. This is probably due to the patient cohort that is offered chemotherapy, which tends to be the more aggressive, triple negative tumors.

### NAT1 expression and response to drug treatment

**Fig. 5:**
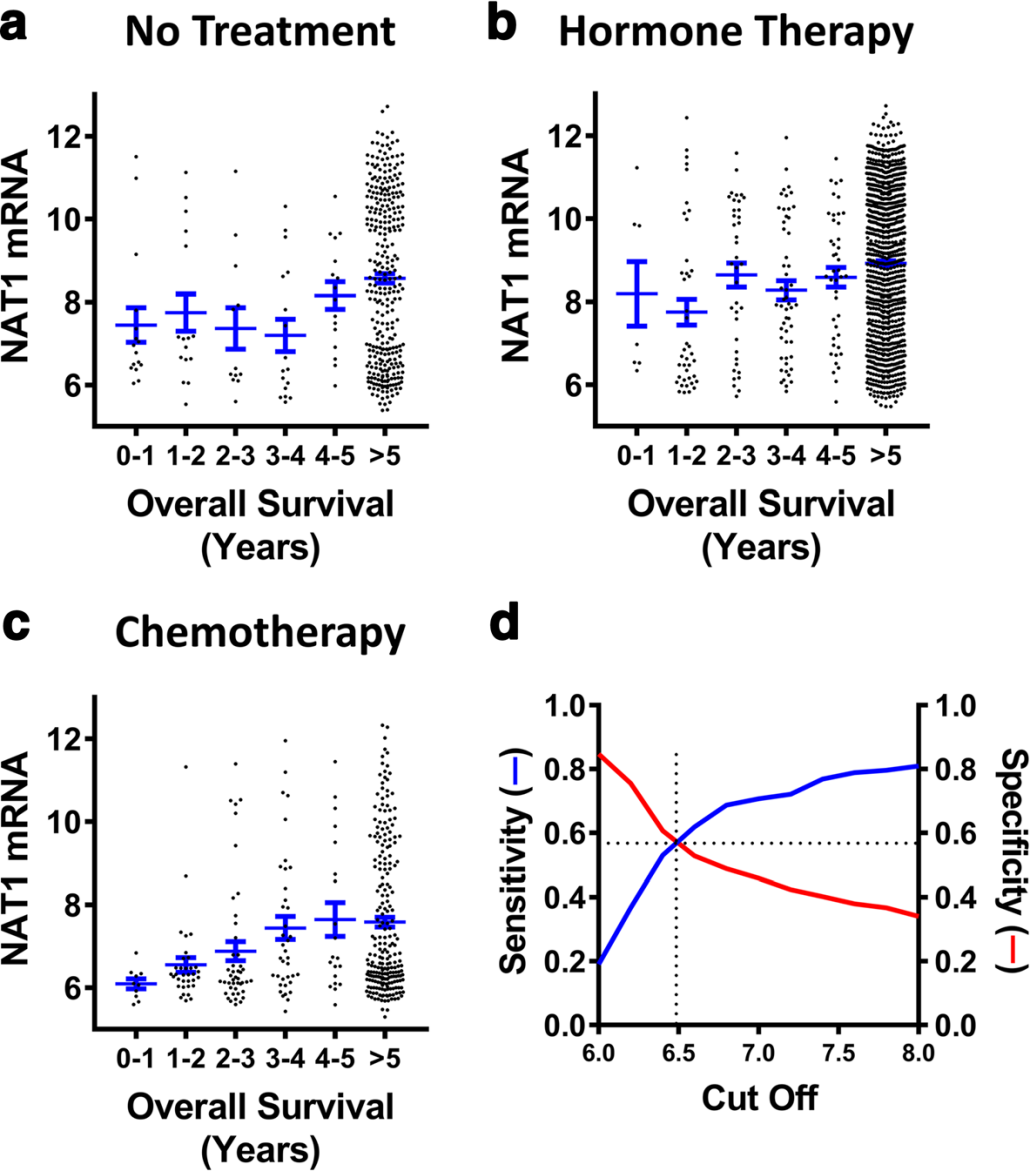
NAT1 expression and response to drug treatment. **a** NAT1 mRNA in patients receiving no drug treatment (*n* = 385) plotted against overall survival of 1 to 5 years or greater than 5 years. **b** NAT1 mRNA in patients receiving hormone therapy (*n* = 1121). **c** NAT1 mRNA in patients receiving chemotherapy (*n* = 375). **d** Decision plot of sensitivity (left axis) and specificity (right axis) for NAT1 mRNA to predict drug resistance (< 5 years survival) or sensitivity (> 5 years survival) against NAT1 mRNA cut-off

There were 541 patients in the METABRIC cohort who received neither hormonal treatment nor chemotherapy. Of these, 385 patients satisfied the inclusion criteria (see Methods). Fig. 5a shows no correlation between survival over the first 5 years and NAT1 mRNA levels in this patient cohort (test for trend *p* = 0.14). When a similar analysis was performed on patients receiving only hormone therapy (*n* = 1121), again no correlation with survival was observed (Fig. 5b; test for trend *p* = 0.08). This result suggests that NAT1 expression does not predict response to hormone therapy. There were 412 patients who received adjuvant chemotherapy with a total of 376 patients who satisfied the inclusion criteria. Figure 5c shows a highly significant correlation between NAT1 expression and survival from years 1 to 5 (Pearson’s *r* = 0.99, test for trend *p* = 0.0008). From the graph, it can be estimated that, during the first 5 years following diagnosis, an increase of one log(2) unit of NAT1 mRNA was associated with an increased survival of 2.4 years. These results show that NAT1 expression predicts survival in those patients who received chemotherapy but not in those who received hormone treatment or no treatment. Early death from the disease following treatment may be a measure of poor response, or chemo-resistance. By contrast, survival beyond 5 years suggests sensitivity to drug treatment. Thus, the results in Fig. 5c suggest that low NAT1 expression is associated with resistance to chemotherapy.

To determine the sensitivity of NAT1 mRNA to predict drug resistance, a decision plot of NAT1 expression levels versus both sensitivity (true positive rate) and specificity (true negative rate) was constructed (Fig. 5d). The cross-over point of the two curves, seen at a log(2) mRNA of ~ 6.5, estimates optimum sensitivity. This shows that NAT1 gene expression can predict resistance to chemotherapy with a sensitivity and specificity of approximately 58% suggesting NAT1 can modulate drug response, or that it is a surrogate marker for other physiological parameters that determine response.

## Discussion

This is the first study to specifically examine the distribution of NAT1 mRNA in primary breast cancers from a well-characterized and extensively studied patient cohort. The trimodal distribution supports multiple mechanisms for regulating NAT1 expression, which was specific to breast cancer and not seen in other cancers such as prostate, ovarian, cervical or colorectal. In those patients expressing low and high levels of NAT1, there was significant correlation with the expression of many other genes. Somewhat surprisingly, very few were shared between the two groups suggesting different genetic regulatory pathways. In the intermediate group, which comprised 38% of all patients, NAT1 mRNA levels correlated with only 3 other genes (PSD3, SEMG1 and PMP22). This observation suggests that NAT1 mRNA in this group was regulated by non-genetic mechanisms. The stability of mRNA is influenced by a number of processes including miRNA- and lncRNA-mediated degradation, exoribonucleases and RNA binding proteins [31–33]. The NAT1 transcript is a potential target for mir-1290, which is differentially expressed in breast cancer. The target sequence is located 551 bp downstream of the stop codon [34]. The NAT1 gene has 3 potential polyadenylation sites situated 286, 387 and 870 bp downstream of the stop codon [35]. Thus, mir-1290 would only target NAT1 transcripts that utilize the longest 3’UTR. It would be interesting to correlate mir-1290 expression with that for NAT1, especially in the intermediate breast cancer group.

While NAT1 mRNA was high in ER positive samples, its expression did not appear to be regulated by ER. This is supported by studies in breast cancer cell-lines [24]. High NAT1 expression was almost always associated with high ESR1 expression. However, low NAT1 expression was associated with both low and high ESR1 expression. Indeed, the strongest association appeared to be the very low or absence of expression in ER negative tumors. The present study suggests that NAT1 may not be a good marker of ER positive breast cancer, especially when its level of expression is low.

NAT1 mRNA showed a strong correlation with overall survival. Patient tumors with a log(2) NAT1 mRNA greater than 11.5 had a 10 year survival of almost 90%. For those patients with log(2) NAT1 mRNA less than 11.5, 10 year survival decreased to less than 70%. Moreover, in the low patient group (log(2) NAT1 mRNA less than 6.5), 30% of patients survived less than 5 years. There have been a number of other studies that have reported the effects of NAT1 in breast cancer recurrence and overall survival. Endo et al. [34] found no difference in relapse-free survival between high and low NAT1 expression, but did see better survival if the high NAT1 patients were also node-positive. By contrast, Andres et al. [36] showed elevated NAT1 expression was associated with a decreased hazards ratio for both mortality and recurrence. The association between NAT1 expression and overall survival does not distinguish cause and effect. Experiments in cells suggest that low NAT1 should result in a less aggressive, more differentiated phenotype [5, 6, 37]. This is consistent with the increase in bone metastasis for breast carcinomas expressing high levels of NAT1 mRNA. However, it does not account for the low survival in those patients with low NAT1 mRNA. A possible explanation is the effect of NAT1 on chemo-sensitivity and overall response to therapy.

There have been several attempts to identify multiple gene signatures that classify breast cancer sub-types or help predict survival [38–44]. NAT1 has been included in signatures for breast cancer identification and staging [11], as a prognostic marker in male breast cancer [17] and non-triple negative breast cancer [45]. Hatzis et al. [39] found predictive genomic signatures for both chemo-sensitivity and hormone sensitivity in breast cancer suggesting a predisposition to chemotherapy response in the cancer patients. In patients receiving chemotherapy, low NAT1 expression was associated with a significant decrease in survival over the first 5 years following diagnosis. This suggests resistance to the drug treatment as no association was seen in those patients who received hormone therapy. The commonly used cytotoxins for breast cancer include taxanes, anthacyclines, 5-fluorouracil and methotrexate. None of these drugs are substrates for NAT1 indicating that differences in drug sensitivity is unlikely to be related to drug metabolism. Alternatively, NAT1 mRNA levels may be a surrogate marker other physiological parameters that determine drug response.

Other groups have reported a change in drug sensitivity in vitro with over-expression or under-expression of NAT1 [4, 46–48]. The positive relationship between NAT1 expression and survival in those patients receiving chemotherapy may be a significant finding that can be used to identify individuals requiring alternative treatment regimens. However, as a single biomarker, its sensitivity and specificity requires improvement for clinical applications. To achieve this, it will be important to understand how NAT1 expression influences chemo-sensitivity. Moreover, an understanding of the underlying mechanisms that link NAT1 to drug response is required.

## Conclusions

There are multiple populations in breast cancer that can be segregated based on NAT1 mRNA levels. For those patients with low expression, overall survival is significantly less than for those patients with intermediate or high expression. Moreover, low NAT1 expression shows a distinct poor response to chemotherapy. Analysis of NAT1 expression may be useful in the future for identifying specific individuals who would benefit from alternative treatments.

## Methods

### Patient microarray and clinical data

All gene expression data were obtained through cBioPortal for Cancer Genomics (http://www.cbioportal.org). A description of the array data methodology and ethical approval for each of the studies is included in the original publications [25, 49–53]. The study was also approved by the Institutional Human Ethics Committee (Approval 2017001552). For RNA expression, data from the METABRIC cohort (*n* = 1980) [49] and The Cancer Genomics Atlas cohort (TCGA; *n* = 1100) [25] were used. Clinical data for the METABRIC patients were also accessed through cBioPortal. For NAT1 expression in ovarian, prostate, cervical and colorectal cancers, data were obtained from TCGA [50–53]. All RNA levels were normalized by log(2) transformation before analysis.

### Source data analysis

Probit analysis, frequency distributions and statistical analyses for specific genes were performed using Prism software (Graphpad Software, La Jolla, USA). For modelling of the frequency distribution of NAT1 transcripts, log(2) transformed values were binned using a bin size of 0.2. Multiple Gaussian distributions (*n* = 1 to 3) were fitted to the frequency data by nonlinear regression using the following equation:

$$ y=\sum \limits_{i=1}^n\left[{A}_i.{e}^{\Big(-0.5.{\left(\frac{\Big(x-{\overline{x}}_i}{SD_i}\right)}^2}\right] $$

where y = observed number in each bin, A = amplitude, $\overline{\mathrm{x}}$ = bin mean, SD = standard deviation and *n* = number of Gaussian distributions. Convergence was confirmed using at least 3 independent initial estimates of each parameter. The area under each Gaussian distribution (AUC) was calculated using:

$$ {AUC}_i={A}_i.{SD}_i.\sqrt{2\pi } $$

Convergence was only observed with a trimodal Gaussian distribution where the correlation coefficient between the observed and predicted values was greater than 0.96 and the standard deviation of the residuals (Sy.x) was 5.74.

### Gene expression correlations

Patients were divided into low, intermediate and high NAT1 expression using log2 cut-off boundaries from 5 to 6.5, 7.25–8.5 and 10–13, respectively. These were chosen to maximise the number of patients in each group while limiting the number of patients incorrectly allocated due to the overlapping Gaussian distributions. This is shown in Additional file 6: Figure S5 where the individual Gaussian curves, along with the selected cut-off boundaries are illustrated. The estimated number of incorrectly assigned patients was 14.8, 1.9 and 4.1% in the low, intermediate and high NAT1 sub-populations, respectively.

To identify genes in each sub-population that co-expressed with NAT1, log(2) transformed mRNA levels for each gene on the Affimetrix array were compared with that for NAT1 using Pearson’s correlation coefficient. Global significance was assumed at *p* < 0.01. Bonferonni’s correction was used for multiple comparisons such that the gene-level significance was *p* < 4 × 10^− 7^. Correlated genes were classified into their predicted molecular functions using the Panther software [26]. The same software was used to identify over-representation of molecular function datasets.

### Breast cancer patient survival analysis

Overall survival of cancer patients in each NAT1 sub-population was analyzed by Kaplan-Meier plots using Prism software (Graphpad Software, La Jolla, USA). Significant differences were assessed with the Log-rank Mantel-Cox test.

### Response to chemotherapy

The METABRIC cohort was divided into patients who received no treatment, patients who received hormone treatment only and patients who received chemotherapy only. Those patients with survival less than 5 years were classified as ‘resistant’ to treatment while those patients with survival longer than 5 years were classified as ‘sensitive’ to treatment. Patients who died of causes other than their cancer within 5 years of diagnosis were excluded from the analysis. In addition, patients who were still alive but their last follow-up was less than 5 years since diagnosis were also excluded (there were no data on whether these individuals survived longer than 5 years). NAT1 expression was then compared for each treatment group versus overall survival time by one-way ANOVA. Specificity and sensitivity calculations for NAT1 mRNA levels and overall survival were performed as described elsewhere [54].

### Hazard ratio estimates

Univariate and multivariate Cox proportional hazard models were applied to identify risk factors for overall survival. The hazard ratio (HR) and 95% confidence intervals (CI) were estimated using SPSS statistical software, version 24.0 (IBM Corp, Armonk, NY). A two-sided *p*-value less than 0.05 was considered statistically significant.

## Additional files

Additional file 1: **Figure S1.** Probit plots of NAT1 mRNA expression from the TCGA provisional database (http://www.cbioportal.org) and from Ciriello et al. Cell 163: 506–519, 2015. (TIF 1303 kb)

Additional file 2: **Table S1.** List of genes that correlate with NAT1 expression in the METABRIC database. (PDF 196 kb)

Additional file 3: **Figure S2.** NAT1 mRNA expression and clinical attributes in the Metabric database (PAM50 and claudin-low populations, Nottinham’s Prognostic Index, histological grade, tumor stage, age at diagnosis and tumor size). (TIF 2405 kb)

Additional file 4: **Figure S3.** Frequency distribution of SRY mRNA in the METABRIC database. **Figure S4.** Kaplan-Meier curves for low and high NAT1 expression using data from Gyorffy et al. (Breast Cancer Res Treat 123: 725–731, 2010). (TIF 4197 kb)

Additional file 5: **Table S2.** The Cox proportional hazard regression models based on survival for NAT1 sub-populations, tumour characteristics and treatment. (PDF 121 kb)

Additional file 6. **Figure S5.** Separation of patients into low, intermediate and high NAT1 expression based on log(2) mRNA levels. (TIF 3115 kb)

## Abbreviations

ER: Estrogen receptor
ESR1: Estrogen receptor gene
NAT1: Arylamine N-acetyltransferase 1
