# Supplementary figures and images for: Trimodal distribution of arylamine N-acetyltransferase 1 mRNA in breast cancer tumors: association with overall survival and drug resistance
Source: BMC Genomics. 2018 Jul 3;19:513. doi: 10.1186/s12864-018-4894-4 (PMC6029418; doi:10.1186/s12864-018-4894-4)

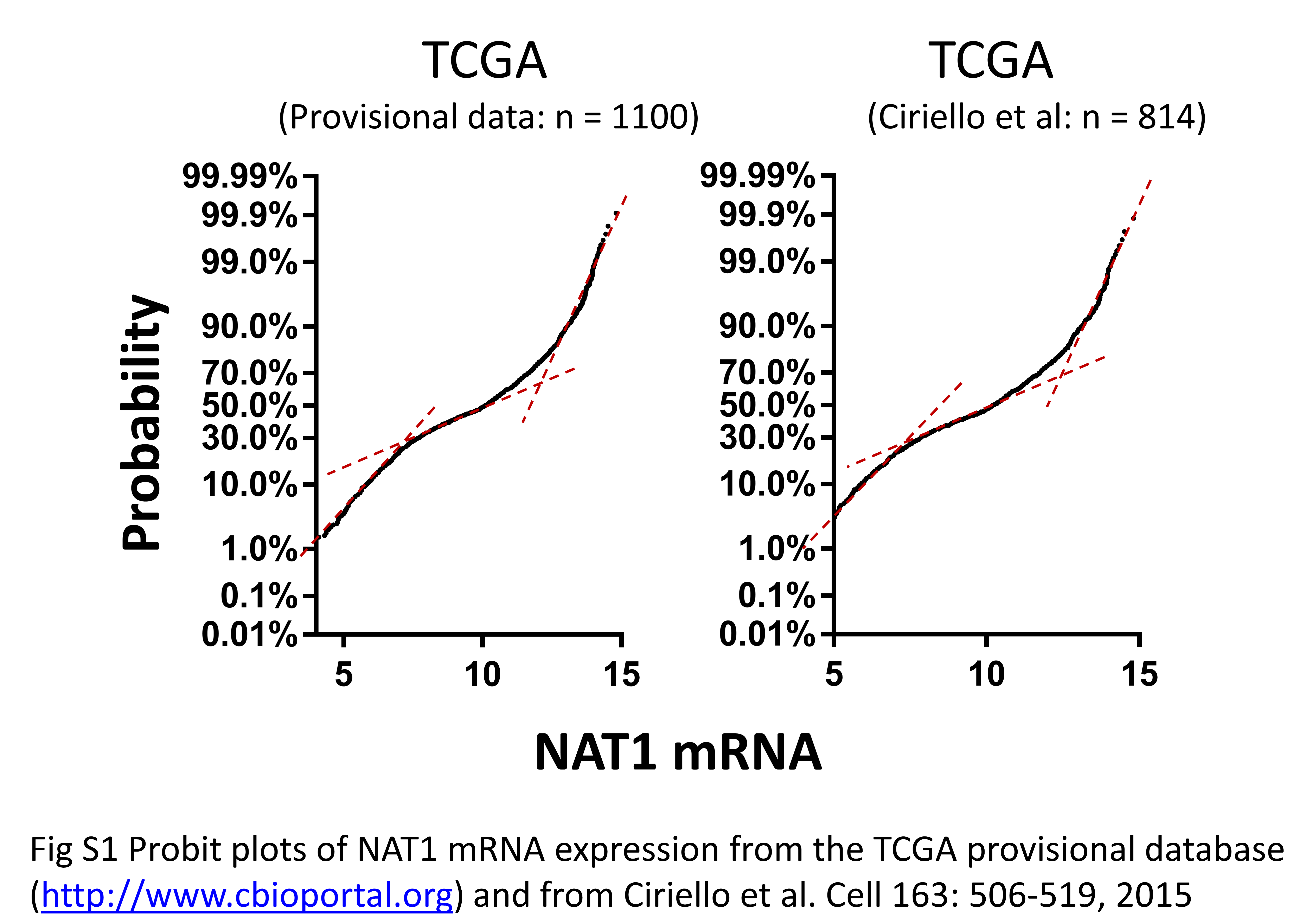

Supplement: Supplementary file 1 — Figure S1. Probit plots of NAT1 mRNA expression from the TCGA provisional database (http://www.cbioportal.org) and from Ciriello et al. Cell 163: 506–519, 2015. (TIF 1303 kb) [file 12864_2018_4894_MOESM1_ESM.tif]

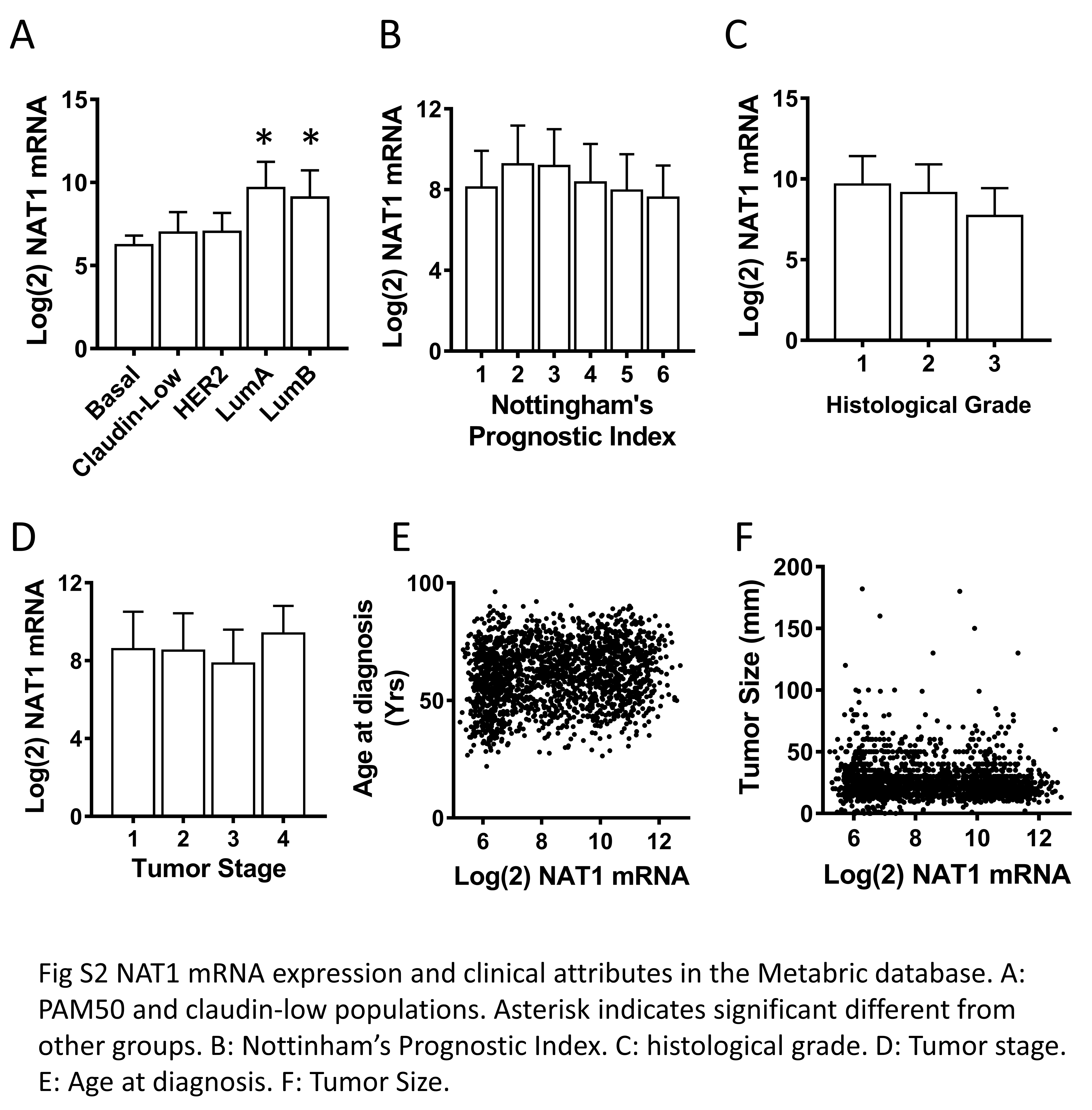

Supplement: Supplementary file 3 — Figure S2. NAT1 mRNA expression and clinical attributes in the Metabric database (PAM50 and claudin-low populations, Nottinham’s Prognostic Index, histological grade, tumor stage, age at diagnosis and tumor size). (TIF 2405 kb) [file 12864_2018_4894_MOESM3_ESM.tif]

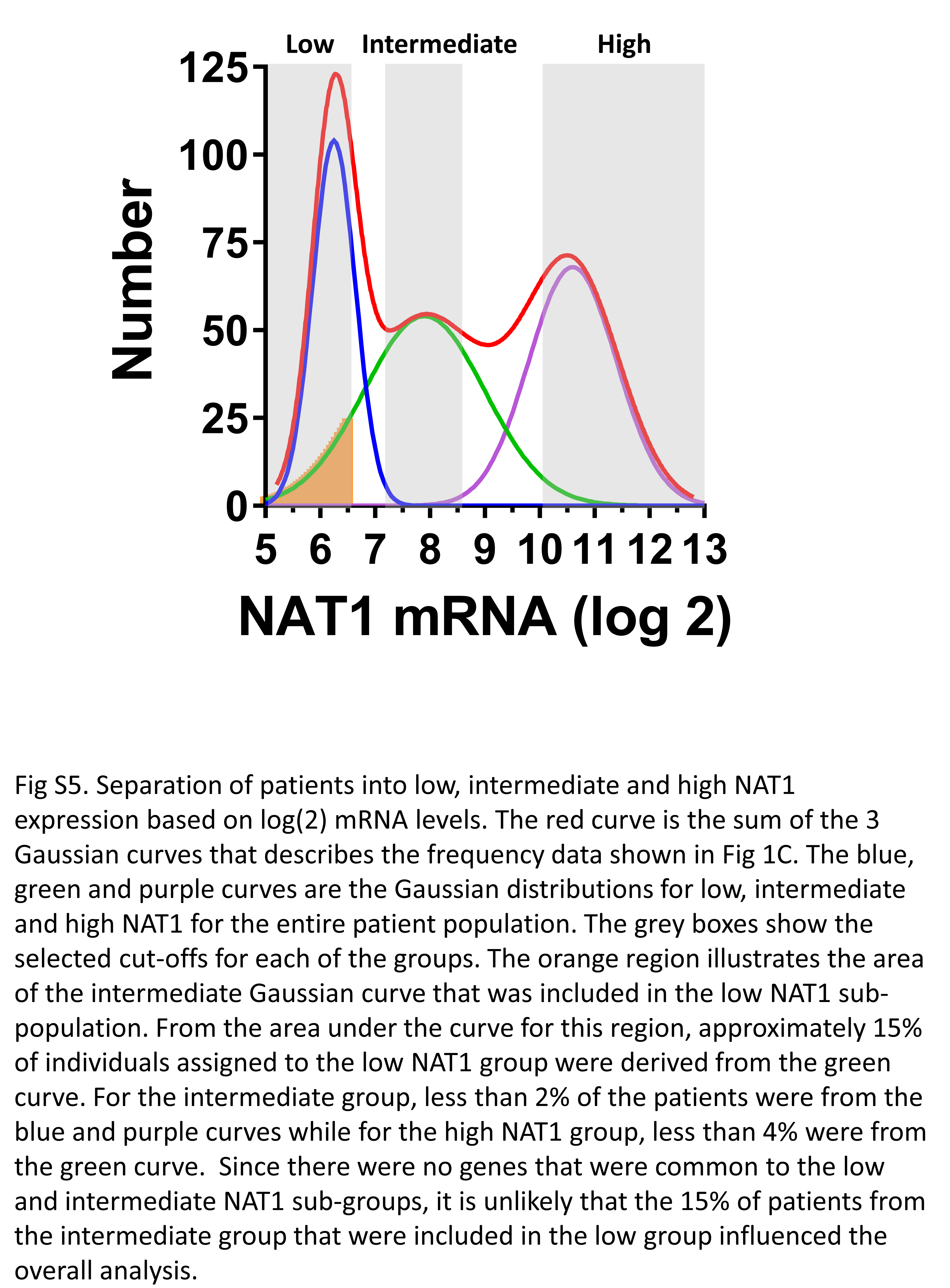

Supplement: Supplementary file 6 — Figure S5. Separation of patients into low, intermediate and high NAT1 expression based on log(2) mRNA levels. (TIF 3115 kb) [file 12864_2018_4894_MOESM6_ESM.tif]
