# Supplementary material for: Trimodal distribution of arylamine N-acetyltransferase 1 mRNA in breast cancer tumors: association with overall survival and drug resistance
Source: BMC Genomics. 2018 Jul 3;19:513. doi: 10.1186/s12864-018-4894-4 (PMC6029418; doi:10.1186/s12864-018-4894-4)
Supplement: Supplementary file 2 — Table S1. List of genes that correlate with NAT1 expression in the METABRIC database. (PDF 196 kb) [file 12864_2018_4894_MOESM2_ESM.pdf]

Table S1 - List of genes that correlate with NAT1 expression in the METABRIC database. +/- depicts whether correlation is positive or negative

Low NAT1			High NAT1			Intermediate NAT1		
Gene	p	+/-	Gene	p	+/-	Gene	p	+/-
TMBIM4	3.991E-21	+	SEMG1	5.263E-88	+	PSD3	2.996E-08	+
PAPD4	4.238E-21	+	PSD3	1.762E-26	+			
RALB	2.903E-20	+	SOX14	2.25E-18	+			
TCF20	6.277E-20	-	CNOT7	3.117E-18	+			
CCNDBP1	6.66E-19	+	TMEM66	5.016E-18	+			
ATP6V0E1	1.833E-18	+	PCM1	6.223E-18	+			
SNRPA	6.169E-18	-	FBXO16	3.461E-17	+			
LIMA1	1.269E-17	+	MTUS1	3.345E-16	+			
RPS27L	3.177E-17	+	DCTN6	2.544E-15	+			
DAZAP2	9.187E-17	+	ZBTB41	3.563E-15	+			
ATN1	1.057E-16	-	BNIP3L	5.207E-15	+			
RPL36AL	1.422E-16	+	C6orf97	8.009E-15	+			
ANKMY2	2.445E-16	+	MRPS14	4.073E-14	+			
COMMD10	2.905E-16	+	ASAH1	7.223E-14	+			
ELF1	3.703E-16	+	DYNC2LI1	2.563E-13	+			
EIF2C2	4.067E-16	-	AGR3	3.398E-13	+			
LRFN4	5.391E-16	-	LOC100506668	3.73E-13	+			
AFF1	7.121E-16	+	CRH	1.414E-12	+			
JKAMP	8.4E-16	+	PRDX3	2.33E-12	+			
MFAP1	9.702E-16	+	PHOSPHO2	2.536E-12	+			
FNIP1	1.333E-15	+	SRP9	3.733E-12	+			
FBXL5	1.878E-15	+	ALG13	5.212E-12	+			
ARPC3	2.628E-15	+	BCLAF1	6.557E-12	+			
ZNF462	2.667E-15	-	LOC729852	6.636E-12	+			
GOLGA5	3.692E-15	+	XPO7	9.913E-12	+			
XBP1	4.062E-15	+	TNKS	1.044E-11	+			
HNMT	4.253E-15	+	CD46	1.068E-11	+			
C14orf129	4.352E-15	+	IKZF5	1.748E-11	+			
SNX2	6.463E-15	+	ALG14	1.759E-11	+			
SH3BGRL	7.091E-15	+	DMD	2.176E-11	+			
PQLC3	9.642E-15	+	ERI2	2.265E-11	+			
ATXN7L2	1.256E-14	-	FBXO3	2.335E-11	+			
BF509118	1.312E-14	-	SRR	3.831E-11	+			
AK124143	1.614E-14	+	PRPF31	4.951E-11	-			
RGMA	1.615E-14	-	CLASP2	6.651E-11	+			
TBC1D22B	1.714E-14	-	ZG16B	6.816E-11	+			
LACTB	1.749E-14	+	GIN1	6.877E-11	+			
SAR1B	1.807E-14	+	EPHX2	6.966E-11	+			
MICALL1	1.833							

GALNT10	2.518E-14	+	ACAT1	8.36E-11	+
SMO	4.288E-14	-	TMCO1	1.36E-10	+
AHDC1	4.966E-14	-	GNG12	1.515E-10	+
CAB39	5.284E-14	+	IQCK	1.556E-10	+
MGST2	5.783E-14	+	CTSA	1.81E-10	-
URB2	6.137E-14	-	CCDC126	2.164E-10	+
TIMM44	8.845E-14	-	CASC1	2.228E-10	+
C4orf34	9.561E-14	+	GTF2H5	3.404E-10	+
VPS29	1.106E-13	+	ZNF627	6.472E-10	+
CDK18	1.148E-13	-	SLC10A3	7.198E-10	-
ACTR10	1.223E-13	+	ST8SIA6	7.773E-10	+
FOXC1	1.297E-13	-	IFT46	7.903E-10	+
GPR137B	1.724E-13	+	SYPL1	8.519E-10	+
SETD1A	1.788E-13	-	RSBN1L	8.572E-10	+
PELP1	2.203E-13	-	ENSA	9.145E-10	+
RBL2	2.328E-13	+	AGPAT5	9.606E-10	+
AP3B1	2.652E-13	+	APITD1	1.025E-09	+
COPS4	2.773E-13	+	C9orf116	1.066E-09	+
SNAP23	2.997E-13	+	C14orf45	1.152E-09	+
GPR125	3.079E-13	-	FOXA1	1.172E-09	+
STRN3	3.186E-13	+	CLUAP1	1.242E-09	+
NARF	3.391E-13	-	CDK14	1.286E-09	+
YPEL5	3.444E-13	+	STXBP3	1.577E-09	+
PIF1	3.526E-13	-	LYPLAL1	1.591E-09	+
RAB40B	3.671E-13	-	ZNF429	1.807E-09	+
ETFA	3.789E-13	+	MRPL22	1.981E-09	+
GIN1	4.304E-13	+	SLC25A1	1.987E-09	-
IL6ST	4.52E-13	+	C10orf57	2.021E-09	+
TCF7L1	4.626E-13	-	FBXO4	2.274E-09	+
BCL11A	4.704E-13	-	UBA3	2.6E-09	+
HEXB	4.733E-13	+	NKIRAS2	2.607E-09	-
EHMT2	5.096E-13	-	C6orf130	2.904E-09	+
ALKBH1	5.144E-13	+	DDX39A	2.988E-09	-
RB1	5.164E-13	+	EXOSC1	3.149E-09	+
MR1	5.581E-13	+	ZNF239	3.79E-09	+
ANG	5.596E-13	+	TRMT1L	3.813E-09	+
ZCCHC9	6.15E-13	+	PIP5K1A	4.3E-09	+
JMJD4	6.298E-13	-	FAM125B	4.461E-09	-
MCMBP	6.702E-13	-	RPAP3	4.758E-09	+
SLC35F5	7.601E-13	+	PTPN7	4.845E-09	-
ALKBH2	7.687E-13	-	TMF1	5.296E-09	+
SEPP1	7.701E-13	+	CR614803	5.337E-09	+
KLHDC3	8.563E-13	-	LIME1	5.514E-09	-
LIPA	8.748E-13	+	COMMD10	5.598E-09	+
TGFBR2	8.971E-13	+	NOTCH2NL	5.786E-09	+
CSNK1E	9.039E-13	-	IFNAR2	5.918E-09	-

SPPL2A	1.223E-12	+	ATG9A	6.032E-09	-
ZNF618	1.23E-12	-	SEH1L	6.172E-09	+
ATP6V0D1	1.281E-12	+	THAP1	6.685E-09	+
SRP54	1.292E-12	+	ACTN4	7.633E-09	-
MORC2	1.405E-12	-	CYB5A	7.868E-09	+
MREG	1.424E-12	+	DYRK2	8.022E-09	-
HSD17B11	1.449E-12	+	PLEKHA3	8.038E-09	+
HERPUD1	1.573E-12	+	SPATA17	8.093E-09	+
CGRRF1	1.591E-12	+	CD2AP	8.107E-09	+
SERPINB1	1.665E-12	+	ELF1	8.62E-09	+
LUM	1.669E-12	+	MGAT1	9.186E-09	-
SLC36A4	1.754E-12	-	YEATS2	9.336E-09	-
CASC4	1.76E-12	+	PDE6B	9.422E-09	+
CYB5A	1.789E-12	+	NUP35	9.631E-09	+
C10orf26	1.829E-12	+	LRRC42	9.982E-09	+
RABL3	2.101E-12	+	C19orf48	1.033E-08	-
TMEM201	2.334E-12	-	AUH	1.036E-08	+
ISCU	2.41E-12	+	TCF4	1.047E-08	-
LMO4	2.536E-12	-	HAUS1	1.095E-08	+
B2M	2.627E-12	+	METTL18	1.1E-08	+
C6orf106	2.685E-12	-	TMEM59	1.175E-08	+
STOX2	2.777E-12	-	NEDD1	1.197E-08	+
RAVER1	2.783E-12	-	NFYA	1.268E-08	+
PLEKHB1	2.809E-12	-	TMEM116	1.335E-08	+
RIOK2	2.826E-12	+	SLC25A24	1.361E-08	+
TPK1	3.334E-12	+	BCDIN3D	1.373E-08	+
PIGB	3.483E-12	+	FYN	1.39E-08	-
SNX30	3.514E-12	+	DDR2	1.424E-08	-
PPRC1	3.546E-12	-	HSPB2-C11orf52	1.454E-08	+
RAD54L	3.621E-12	-	SDHC	1.509E-08	+
TM6SF1	3.688E-12	+	LRRC6	1.592E-08	+
GIT1	3.854E-12	-	RSPH3	1.604E-08	+
SSBP3	4.014E-12	-	MYO1G	1.653E-08	-
KIAA1647	4.243E-12	-	UBR1	1.711E-08	+
CEP164	4.534E-12	-	PLBD2	1.803E-08	-
TOMM40	4.605E-12	-	MYNN	1.813E-08	+
CHRM3	5.051E-12	-	TMEM14A	1.824E-08	+
PHLPP1	5.117E-12	-	GK	1.866E-08	+
ARHGEF11	5.246E-12	-	SH3BP1	2.094E-08	-
UBE2L6	6.128E-12	+	DTWD1	2.137E-08	+
CSNK1A1	6.183E-12	+	CAPN7	2.177E-08	+
SAMD4B	6.361E-12	-	SLC35C2	2.224E-08	-
ATG9A	6.421E-12	-	FAM164A	2.308E-08	-
Tmp21-II	6.533E-12	+	ARV1	2.371E-08	+
KCNK5	6.739E-12	-	IDE	2.38E-08	+
MLLT4	6.82E-12	-	SCO1	2.396E-08	+

SLMO1	7.038E-12	-	UFC1	2.429E-08	+
AGGF1	7.365E-12	+	NCKAP1	2.511E-08	+
NT5DC2	7.674E-12	-	HAPLN3	2.744E-08	-
MCM3APAS	8.191E-12	-	SNX13	2.849E-08	+
TCEAL1	8.419E-12	+	FAM38A	2.87E-08	-
ARMCX3	8.577E-12	+	TMEM56	2.915E-08	+
ITM2B	8.681E-12	+	YTHDF1	2.931E-08	-
USP21	9.229E-12	-	CYB5R3	2.955E-08	-
TSC22D3	9.402E-12	+	OGFR	3.028E-08	-
TMEM188	1.017E-11	+	NME7	3.103E-08	+
SSRP1	1.07E-11	-	MATR3	3.154E-08	+
HGS	1.089E-11	-	IP6K2	3.581E-08	+
SPTBN2	1.148E-11	-	DYNLL1	3.814E-08	+
FBXO4	1.226E-11	+	MR1	3.874E-08	+
ALOX5AP	1.228E-11	+	DHX9	4.004E-08	+
LOC161527	1.236E-11	-	GGPS1	4.079E-08	+
CYB561D2	1.248E-11	+	ESR1	4.092E-08	+
SUPT5H	1.275E-11	-	PSMC6	4.159E-08	+
NDUFS4	1.335E-11	+	SCP2	4.29E-08	+
KDM6A	1.423E-11	+	MIOS	4.587E-08	+
LOC729852	1.475E-11	+	DAZAP2	4.617E-08	+
TNPO2	1.478E-11	-	DHX38	4.628E-08	-
TMTC3	1.524E-11	+	MAN2B1	4.675E-08	-
PNRC2	1.567E-11	+	TMBIM4	4.687E-08	+
SPCS1	1.729E-11	+	UBE2G1	4.924E-08	+
DAD1	1.736E-11	+	PNRC2	4.98E-08	+
ZFAND6	1.741E-11	+	STAM2	5.322E-08	+
PPP1R9A	1.754E-11	-	KIF5B	5.544E-08	+
C9orf152	1.823E-11	+	BCCIP	6.017E-08	+
CTIF	1.845E-11	-	ELMOD2	6.214E-08	+
PTGR1	1.869E-11	+	IFT88	6.912E-08	+
FAM174A	1.918E-11	+	FBXO31	7.15E-08	-
ELOVL5	1.928E-11	+	KBTBD3	7.177E-08	+
ITFG1	2.089E-11	+	XBP1	7.254E-08	+
SLC40A1	2.092E-11	+	RC74	7.377E-08	+
TBC1D9	2.108E-11	+	SPA17	7.562E-08	+
MTUS1	2.157E-11	+	SRGAP1	7.598E-08	-
POLR1E	2.161E-11	-	VASN	8.049E-08	-
AK023756	2.162E-11	-	PPP6R1	8.328E-08	-
C15orf42	2.167E-11	-	VPS29	8.404E-08	+
CHMP5	2.191E-11	+	UBE3A	8.553E-08	+
CD46	2.242E-11	+	CWF19L2	8.93E-08	+
AK129717	2.303E-11	-	ATPAF1	8.989E-08	+
OBSCN	2.355E-11	-	RAB4A	9.299E-08	+
FURIN	2.382E-11	-	CD7	9.305E-08	-
SAMD1	2.503E-11	-	UNC119B	9.473E-08	+

MRPL2	2.71E-11	-	TRIM13	9.593E-08	+
GTSE1	2.711E-11	-	ARFGAP1	9.639E-08	-
MTPAP	2.785E-11	-	RBM42	1.011E-07	-
FAM82A2	2.821E-11	+	ACADM	1.025E-07	+
CTDP1	2.882E-11	-	SSR2	1.053E-07	+
LPAR6	2.898E-11	+	NUDT18	1.065E-07	+
PPP1R2	3.004E-11	+	TPRKB	1.079E-07	+
MORF4L2	3.186E-11	+	ADAM10	1.128E-07	+
LOC390705	3.399E-11	-	TMC6	1.226E-07	-
PSMA6	3.519E-11	+	CLUL1	1.314E-07	+
OSTF1	3.533E-11	+	PDPR	1.315E-07	-
FAM161A	3.601E-11	-	RABL3	1.334E-07	+
PPP2R5D	3.895E-11	-	CD4	1.348E-07	-
IL13RA1	4.051E-11	+	TMEM69	1.351E-07	+
ANAPC13	4.064E-11	+	SPATA4	1.354E-07	+
FANCE	4.219E-11	-	ITM2C	1.361E-07	-
AP3S1	4.312E-11	+	KIAA1737	1.381E-07	+
PLIN3	4.357E-11	+	MAP4K1	1.438E-07	-
TRAF2	4.361E-11	-	CLDND2	1.458E-07	-
DSCR3	4.507E-11	+	BTBD1	1.473E-07	+
BLM	4.525E-11	-	BCOR	1.496E-07	+
SF3A3	4.591E-11	-	ZNF30	1.518E-07	+
TTC8	4.65E-11	+	UBA5	1.575E-07	+
ProSAPiP1	4.703E-11	-	WRN	1.674E-07	+
PIGF	4.761E-11	+	ZNF484	1.675E-07	+
FAM171A1	4.992E-11	-	APIP	1.695E-07	+
TCF7L2	5.002E-11	-	PGM2	1.713E-07	+
FBXO8	5.373E-11	+	SYK	1.762E-07	-
TMEM131	5.402E-11	+	GORAB	1.775E-07	+
IPO13	5.537E-11	-	UCHL3	1.814E-07	+
NRTN	5.561E-11	-	UNC50	1.833E-07	+
PRRC2A	5.993E-11	-	RIT1	1.857E-07	+
TMEM192	6E-11	+	LRRC40	1.902E-07	+
MCM10	6.031E-11	-	GJA1	1.952E-07	+
PLEKHF2	6.048E-11	+	ZMYND12	2.094E-07	+
WDR74	6.103E-11	-	ENPP4	2.097E-07	+
PSMC6	6.184E-11	+	MRAS	2.106E-07	-
SEC23B	6.558E-11	+	SEC23IP	2.165E-07	+
FBXO41	6.905E-11	-	STT3A	2.225E-07	+
SNAPC4	6.985E-11	-	SPOCK2	2.228E-07	-
MYLIP	7.084E-11	+	PIGB	2.265E-07	+
UBE2O	7.334E-11	-	JF432672	2.295E-07	+
MYOF	7.712E-11	+	PLCG1	2.298E-07	-
PRDX3	7.733E-11	+	TMEM86B	2.367E-07	-
BC038245	7.988E-11	-	MITD1	2.397E-07	+
SQRDL	8.169E-11	+	SYF2	2.514E-07	+

POLE	8.528E-11	-	WARS	2.535E-07	-
PPARD	8.639E-11	-	NARF	2.551E-07	-
LZTS2	8.907E-11	-	NSMCE4A	2.551E-07	+
C4orf32	8.917E-11	+	CELF1	2.674E-07	+
MGAT2	8.98E-11	+	GHITM	2.676E-07	+
MED7	9.29E-11	+	DIXDC1	2.69E-07	+
GPR34	9.301E-11	+	EFNB1	2.709E-07	-
ARFIP1	9.342E-11	+	RBBP4	2.715E-07	+
SF3B3	9.445E-11	-	PPWD1	2.844E-07	+
ANKRA2	9.547E-11	+	CASD1	2.905E-07	+
PPP2CA	9.653E-11	+	AP1M1	2.938E-07	-
GPRC5B	9.674E-11	-	FAM120AOS	2.967E-07	+
DNA2	9.807E-11	-	POLR2B	2.979E-07	+
LEO1	1.017E-10	+	EYA2	2.98E-07	-
ALG11	1.026E-10	+	EIF2C2	2.99E-07	-
FAM120AOS	1.028E-10	+	NSUN3	3.042E-07	+
C15orf24	1.037E-10	+	STEAP1	3.206E-07	+
SAC3D1	1.046E-10	-	VPS4A	3.268E-07	-
CHD4	1.098E-10	-	PMPCB	3.271E-07	+
PRRC1	1.107E-10	+	CNIH	3.275E-07	+
ACTN4	1.122E-10	-	PGR	3.299E-07	+
KIF15	1.187E-10	-	MED7	3.386E-07	+
FOXA1	1.21E-10	+	NT5DC2	3.47E-07	-
NOP2	1.239E-10	-	EHF	3.552E-07	+
CHERP	1.282E-10	-	C10orf32	3.603E-07	+
POLR2F	1.302E-10	-	PRKX	3.678E-07	-
RECQL4	1.318E-10	-	GPR89B	3.699E-07	+
TFAP4	1.332E-10	-	UBB	3.772E-07	+
RCOR2	1.352E-10	-	LARP6	3.877E-07	-
C14orf45	1.422E-10	+	ZNF787	3.894E-07	-
AP3M1	1.48E-10	+	AIF1L	3.942E-07	-
ZNF514	1.486E-10	-	HSPB11	3.976E-07	+
NFIX	1.501E-10	-			
TOP1MT	1.509E-10	-			
CRYZL1	1.536E-10	+			
PYGO2	1.602E-10	-			
DNASE1L1	1.642E-10	+			
VOPP1	1.669E-10	+			
DHX33	1.682E-10	-			
MAPK9	1.803E-10	+			
UBC	1.811E-10	-			
USP13	1.828E-10	-			
TLR7	1.879E-10	+			
CMTM1	1.894E-10	+			
TMEM66	1.897E-10	+			
FBXW11	1.921E-10	+			

CNIH	1.943E-10	+
WDR61	1.965E-10	+
DMD	1.967E-10	-
AFG3L2	2.049E-10	-
ARHGEF4	2.127E-10	-
QRICH2	2.204E-10	-
TRIM16	2.249E-10	-
KIAA0664	2.268E-10	-
BMP2K	2.291E-10	+
ZNF248	2.294E-10	-
BLVRA	2.351E-10	+
HPS3	2.409E-10	+
STAG3L2	2.503E-10	-
SNORA10	2.515E-10	-
CTSO	2.571E-10	+
ZNF142	2.586E-10	-
GNL2	2.694E-10	-
PMPCA	2.78E-10	-
RBFOX2	2.811E-10	-
DGAT2	2.849E-10	-
ITPR1	3.003E-10	+
RASD2	3.067E-10	-
COPS7B	3.1E-10	-
EPG5	3.139E-10	-
PPARGC1B	3.142E-10	-
RAB5C	3.26E-10	+
ANKRD13B	3.439E-10	-
EDEM1	3.456E-10	+
VAMP3	3.562E-10	+
AW972870	3.566E-10	-
NFIB	3.58E-10	-
SLC25A37	3.715E-10	-
PLXNC1	3.719E-10	+
GNAS	3.722E-10	-
CAMSAP1L1	3.754E-10	-
CCNG1	3.773E-10	+
INPP4B	3.794E-10	+
DEPDC5	3.808E-10	-
JAK1	3.877E-10	+
G3BP2	3.986E-10	+
ANKRD16	4.038E-10	-
TTK	4.118E-10	-
UBE2A	4.171E-10	+
EPHB3	4.174E-10	-
FOSL1	4.198E-10	-
AK022594	4.263E-10	-

NOP10	4.461E-10	+
CAPN10	4.51E-10	-
SKP2	4.624E-10	-
IDH1	4.636E-10	+
BTF3	4.674E-10	+
TRIP4	4.683E-10	+
TMCO3	4.753E-10	+
C10orf32	4.892E-10	+
PLCB4	4.953E-10	-
ILF3	4.97E-10	-
PRSS53	5.003E-10	-
TULP3	5.017E-10	-
C16orf55	5.045E-10	-
SLU7	5.05E-10	+
NRIP1	5.079E-10	+
PRKX	5.126E-10	-
C5orf32	5.262E-10	+
TMED2	5.432E-10	+
FAM123B	5.503E-10	-
C20orf20	5.503E-10	-
EIF3E	5.523E-10	-
E2F3	5.662E-10	-
NOL9	5.691E-10	-
CD2BP2	5.797E-10	+
SEC23IP	5.888E-10	+
CANX	5.961E-10	+
COL27A1	5.987E-10	-
CADM4	6.036E-10	-
PTPRO	6.052E-10	+
ERI3	6.178E-10	-
MAPK4	6.233E-10	-
C10orf32-AS3	6.239E-10	+
MAN1C1	6.269E-10	+
BYSL	6.399E-10	-
FGFRL1	6.473E-10	-
DPYD	6.629E-10	+
MNDA	6.747E-10	+
SYPL1	6.876E-10	+
WDR5	6.902E-10	-
RILPL2	6.95E-10	+
ZNF318	7.058E-10	-
DQ896551	7.076E-10	+
BQ188538	7.245E-10	-
NRG2	7.544E-10	-
VPS25	7.636E-10	+
SPTLC2	7.891E-10	+

BIRC5	7.965E-10	-
UGT8	8.161E-10	-
MAFB	8.289E-10	+
ETV6	8.337E-10	-
C14orf147	8.339E-10	+
PSMB8	8.417E-10	+
KIFC1	8.43E-10	-
WDR44	8.716E-10	+
CHST3	8.773E-10	-
TMEM85	8.783E-10	+
RPRD2	8.793E-10	-
ELAVL1	8.982E-10	-
FBXO18	9.446E-10	-
TRRAP	9.524E-10	-
CTNNA1	9.625E-10	+
GAS2L3	9.725E-10	-
DTNB	9.895E-10	-
HOXB2	9.959E-10	+
GABBR2	1.017E-09	-
MRPL22	1.034E-09	+
NCS1	1.089E-09	-
CABYR	1.11E-09	-
MLF2	1.147E-09	-
FOXP4	1.158E-09	-
PCYT2	1.205E-09	-
AK125440	1.236E-09	-
GRN	1.281E-09	+
CLASP1	1.299E-09	-
TRAPPC2	1.3E-09	+
ACOT1	1.35E-09	+
PELO	1.357E-09	+
CEP120	1.359E-09	+
STK25	1.391E-09	-
GABARAPL2	1.412E-09	+
HOPX	1.424E-09	+
ZNF692	1.43E-09	-
IGBP1	1.464E-09	+
TNPO3	1.465E-09	-
RABEP1	1.465E-09	+
KHDRBS3	1.474E-09	-
MLPH	1.483E-09	+
AZI1	1.509E-09	-
NUDT3	1.559E-09	-
POM121C	1.571E-09	-
GPRIN2	1.581E-09	-
ARID4A	1.622E-09	+

MS4A6A	1.632E-09	+
RNASE6	1.665E-09	+
RPAP3	1.703E-09	+
TOP3A	1.703E-09	-
GIMAP6	1.738E-09	+
SPG11	1.742E-09	+
GNG10	1.759E-09	+
UBQLN4	1.79E-09	-
FTSJD1	1.815E-09	+
RGS18	1.904E-09	+
NFATC1	1.909E-09	-
KAZN	1.916E-09	-
PI4KB	1.919E-09	-
RIN2	1.96E-09	+
SOX8	1.988E-09	-
MSL3	1.995E-09	-
HSD17B8	2.075E-09	+
TAF6L	2.223E-09	-
PFDN1	2.247E-09	+
MLL4	2.275E-09	-
DARS	2.352E-09	-
DLGAP4	2.372E-09	-
STAM2	2.374E-09	+
TBCA	2.4E-09	+
ZMAT2	2.511E-09	+
NKRF	2.523E-09	-
CPA3	2.558E-09	+
POU2F1	2.601E-09	-
NHLRC3	2.617E-09	+
SOX10	2.72E-09	-
TTYH1	2.771E-09	-
UBL3	2.78E-09	+
LCMT2	2.798E-09	+
ZNF74	2.831E-09	-
PRKCH	2.88E-09	+
EN1	2.884E-09	-
RAB9B	2.924E-09	-
ADCK3	2.939E-09	-
WAPAL	2.959E-09	+
MOCS2	2.964E-09	+
ALDOA	3.023E-09	+
ACOT4	3.042E-09	+
HAUS8	3.062E-09	-
IK	3.126E-09	+
HSD11B1L	3.262E-09	-
HSD17B4	3.284E-09	+

C7orf44	3.327E-09	+
AB074162	3.456E-09	-
C14orf142	3.466E-09	+
BRPF1	3.471E-09	-
DYNC2LI1	3.496E-09	+
POLD1	3.516E-09	-
DHX34	3.535E-09	-
UQCRQ	3.585E-09	+
PIGH	3.628E-09	+
CNTROB	3.703E-09	-
PPME1	3.756E-09	-
RPA3	3.756E-09	+
CDCA3	3.784E-09	-
ATXN7L3	3.787E-09	-
BC009629	3.802E-09	-
FRMD3	3.847E-09	-
ZNF326	3.907E-09	-
CDK19	3.967E-09	-
RAVER2	3.968E-09	-
BNIP3L	3.976E-09	+
PVR	4.213E-09	-
AKR1A1	4.264E-09	+
EIF4G2	4.27E-09	+
PSMC1	4.684E-09	+
UBTD2	4.751E-09	+
GNG11	4.785E-09	+
CYB5R4	4.849E-09	+
CDC25A	4.943E-09	-
OTUB1	4.995E-09	-
EFCAB11	5.017E-09	+
PSMD10	5.051E-09	+
TAF7	5.056E-09	+
SAMSN1	5.064E-09	+
DKFZp761E19	5.065E-09	-
COQ5	5.156E-09	+
MRPL54	5.205E-09	+
ARL2BP	5.29E-09	+
SNRPN	5.312E-09	+
FOXM1	5.373E-09	-
TSPAN13	5.447E-09	+
TMEM50B	5.549E-09	+
SYF2	5.551E-09	+
LAMTOR3	5.696E-09	+
DRAM2	5.701E-09	+
VANGL2	5.711E-09	-
FUBP3	5.753E-09	+

IGFBP7	5.844E-09	+
ROPN1B	5.952E-09	-
CDV3	6.01E-09	+
SEMA6C	6.021E-09	-
ZNF484	6.108E-09	+
AK095715	6.138E-09	-
SIDT1	6.162E-09	+
EXTL3	6.424E-09	-
VAMP5	6.443E-09	+
NSA2	6.486E-09	+
GPR3	6.507E-09	-
NUP188	6.542E-09	-
SPARCL1	6.564E-09	+
CASP8	6.618E-09	+
COX7C	6.621E-09	+
EIF2B2	6.691E-09	+
PTPRE	6.917E-09	+
JAG2	6.977E-09	-
EIF3B	7.065E-09	-
CDH19	7.133E-09	-
AMACR	7.139E-09	+
C20orf30	7.158E-09	+
SLC45A4	7.184E-09	-
TUB	7.242E-09	-
PSME1	7.244E-09	+
ROPN1	7.254E-09	-
KIAA0562	7.441E-09	-
MOSC2	7.492E-09	+
EIF4E	7.541E-09	+
CDC20	7.638E-09	-
RNF4	7.769E-09	+
C10orf128	7.771E-09	+
RBM10	7.851E-09	-
MTF1	7.857E-09	-
BHLHE40	7.885E-09	+
ATP6V1D	8.045E-09	+
B3GAT3	8.11E-09	-
SMG7	8.211E-09	-
NOSTRIN	8.316E-09	+
NEURL4	8.408E-09	-
UNC50	8.415E-09	+
MCM5	8.494E-09	-
BX647938	8.539E-09	-
RNF103	8.691E-09	+
STX8	8.761E-09	+
SAP30BP	8.807E-09	-

MRPS18C	8.896E-09	+
FKRP	8.954E-09	-
CLIP2	9.033E-09	-
SPEG	9.154E-09	-
YTHDC2	9.172E-09	+
DDX51	9.204E-09	-
SLC6A8	9.248E-09	-
PUS7	9.344E-09	-
GMPPB	9.35E-09	+
TMED10	9.403E-09	+
UBR1	9.416E-09	+
CHCHD5	9.423E-09	+
ZNF707	9.732E-09	-
CHMP2A	9.971E-09	+
ARMC6	9.977E-09	-
TBCB	1.005E-08	-
GCNT2	1.005E-08	-
REV1	1.025E-08	+
TBRG4	1.031E-08	-
MARK2	1.036E-08	-
TINF2	1.04E-08	+
FBP1	1.041E-08	+
MIIP	1.049E-08	-
XPO5	1.072E-08	-
DDX39B	1.079E-08	-
F13A1	1.084E-08	+
PDHA1	1.087E-08	-
SRP9	1.096E-08	+
TMCC2	1.1E-08	-
TYRO3	1.104E-08	-
TAF4B	1.119E-08	-
KIAA0494	1.147E-08	+
LGMN	1.152E-08	+
HM13	1.168E-08	+
RTKN	1.188E-08	-
VARS	1.194E-08	-
SLC6A10P	1.202E-08	-
MMS22L	1.205E-08	-
GDPD5	1.214E-08	-
PHKB	1.241E-08	+
TATDN2	1.248E-08	-
ZNF3	1.248E-08	-
ZNF473	1.253E-08	-
MRPL12	1.255E-08	-
BAG6	1.262E-08	-
SKAP2	1.263E-08	+

AK022085	1.271E-08	-
ZNF691	1.273E-08	-
RRP12	1.286E-08	-
ZYG11B	1.313E-08	-
POLD4	1.33E-08	+
NPLOC4	1.34E-08	-
RRM2B	1.363E-08	+
NDUFAF1	1.417E-08	+
PDE9A	1.437E-08	-
ALG1	1.44E-08	+
KIAA0353	1.442E-08	-
CHTF18	1.46E-08	-
DDX60	1.476E-08	+
FAM59B	1.497E-08	-
MRFAP1	1.516E-08	+
ITGAV	1.519E-08	+
PTCD2	1.54E-08	+
BEX4	1.571E-08	+
IRF2BP1	1.576E-08	-
C9orf103	1.576E-08	+
ARHGAP21	1.585E-08	-
PCBP4	1.603E-08	-
HJURP	1.637E-08	-
ZNF574	1.654E-08	-
LOC389906	1.668E-08	-
FBXL3	1.702E-08	+
EIF5	1.722E-08	+
RPRD1A	1.81E-08	-
UBB	1.856E-08	+
LOC647979	1.859E-08	+
CNIH2	1.865E-08	-
CDCA8	1.894E-08	-
AF038185	1.909E-08	-
ZNF330	1.915E-08	+
LLPH	1.932E-08	+
CENPF	1.932E-08	-
ELK1	1.972E-08	-
C14orf135	1.974E-08	+
MNT	1.99E-08	-
LTC4S	1.992E-08	+
PPP2R3B	2.015E-08	-
SRRT	2.043E-08	-
ZNF529	2.064E-08	-
SEPHS1	2.079E-08	-
DQ896666	2.136E-08	+
RBM38	2.166E-08	-

FRMD6	2.244E-08	+
SCMH1	2.25E-08	-
GFM2	2.253E-08	+
YPEL2	2.266E-08	+
FZD9	2.271E-08	-
C17orf53	2.289E-08	-
HDAC5	2.29E-08	-
DCN	2.308E-08	+
RCC2	2.32E-08	-
ALMS1	2.327E-08	-
PPIC	2.334E-08	+
STRN4	2.35E-08	-
DGCR8	2.35E-08	-
MAP4K3	2.355E-08	+
NAA40	2.368E-08	-
ZNF217	2.375E-08	+
NCAN	2.391E-08	-
EPHB1	2.412E-08	-
ATP6V1A	2.419E-08	+
HLA-DRA	2.42E-08	+
ITGB5	2.427E-08	+
NFKB2	2.475E-08	-
NFE2L2	2.494E-08	+
MSR1	2.517E-08	+
SC5DL	2.537E-08	+
NPTN	2.566E-08	+
SERF2	2.593E-08	+
C6orf26	2.596E-08	-
POLR3GL	2.597E-08	+
PPP1R3C	2.604E-08	+
CCDC77	2.617E-08	-
STARD3NL	2.64E-08	+
NCEH1	2.681E-08	+
KDM1A	2.707E-08	-
TRMT61A	2.749E-08	-
EEF1A1	2.758E-08	+
AL109696	2.781E-08	-
MIOS	2.797E-08	+
C19orf48	2.797E-08	-
SLC35A3	2.807E-08	+
MDP1	2.823E-08	+
MAP3K11	2.883E-08	-
TGOLN2	2.923E-08	+
RARRES3	2.995E-08	+
ASAH1	3.016E-08	+
TTLL4	3.042E-08	-

ZDHHC8	3.077E-08	-
KIF2C	3.093E-08	-
AGAP3	3.134E-08	-
SERPINA1	3.154E-08	+
KBTBD4	3.217E-08	+
AIF1	3.232E-08	+
ZMYND19	3.289E-08	-
VPS41	3.289E-08	+
BAIAP2L2	3.316E-08	-
CACNA1A	3.347E-08	-
PNPLA6	3.376E-08	-
TMX1	3.405E-08	+
RSPO2	3.405E-08	-
COX17	3.505E-08	+
C5orf15	3.509E-08	+
LYG1	3.514E-08	-
LMBRD1	3.537E-08	+
UPF1	3.546E-08	-
PAIP2	3.574E-08	+
CXXC1	3.584E-08	-
P/OKcl.19	3.706E-08	-
DIS3L2	3.724E-08	-
SETDB1	3.773E-08	-
SUPV3L1	3.801E-08	-
SPTBN5	3.809E-08	-
HIPK2	3.864E-08	-
STOM	3.967E-08	+
SH3GL3	3.974E-08	-
POSTN	3.984E-08	+
GATM	3.99E-08	+
ASB6	4.015E-08	-
USP53	4.051E-08	+
C5orf44	4.226E-08	+
PLCH1	4.266E-08	-
AHNAK	4.27E-08	+
SMYD5	4.295E-08	-
KLC2	4.329E-08	-
ASXL1	4.361E-08	-
ATP5J	4.366E-08	+
NDUFA12	4.444E-08	+
SNORA64	4.473E-08	-
CCNL2	4.556E-08	+
GMPR2	4.562E-08	+
PIGP	4.599E-08	+
SFRP4	4.626E-08	+
ARF4	4.681E-08	+

AFF4	4.687E-08	+
ARL5B	4.753E-08	-
EID1	4.791E-08	+
MAP1S	4.796E-08	-
HLA-DPA1	4.798E-08	+
SEC61G	4.875E-08	+
SCHIP1	4.885E-08	-
CX3CR1	4.891E-08	+
FAM100A	4.914E-08	-
FAM90A7	4.964E-08	-
CRTAP	4.983E-08	+
PCCA	5.019E-08	+
IL10RB	5.051E-08	+
SUCLA2	5.067E-08	+
RUNX2	5.085E-08	+
C18orf56	5.087E-08	-
RAB27A	5.099E-08	+
ZNF282	5.126E-08	-
STIL	5.21E-08	-
ATP5F1	5.21E-08	+
LAMP2	5.323E-08	+
TRIM5	5.4E-08	+
PEX2	5.404E-08	+
PLOD1	5.423E-08	-
UBE2D3	5.442E-08	+
CNKSR3	5.449E-08	-
ACVR2B	5.562E-08	-
NDUFB3	5.585E-08	+
SLC31A2	5.628E-08	+
ZNF143	5.666E-08	+
DAXX	5.678E-08	-
USP5	5.7E-08	-
FAM89B	5.72E-08	-
PIGT	5.737E-08	+
SPCS2	5.751E-08	+
C16orf5	5.792E-08	-
AARS2	5.793E-08	-
CNOT10	5.829E-08	-
PPP1R12B	5.831E-08	+
P53TG1	5.901E-08	+
SEC22C	5.959E-08	+
SLC6A15	5.959E-08	-
HMGN1	6.005E-08	+
SNORD38A	6.039E-08	-
HGC6.4	6.083E-08	-
TMED5	6.091E-08	+

ZNF512B	6.12E-08	-
GNG12	6.165E-08	+
FCHO2	6.198E-08	+
RNF220	6.216E-08	-
ATP7A	6.242E-08	+
KLHL17	6.332E-08	-
IQCE	6.332E-08	-
INO80B	6.36E-08	-
GIGYF1	6.382E-08	-
VWA5A	6.385E-08	+
C11orf95	6.449E-08	-
FMO6P	6.512E-08	-
ZNF552	6.572E-08	+
MGAT4A	6.583E-08	+
RIMS3	6.605E-08	-
FBXO3	6.643E-08	+
PA2G4	6.682E-08	-
SUSD3	6.692E-08	+
EIF4E3	6.697E-08	+
SH2B3	6.739E-08	+
NR2C2AP	6.874E-08	-
DMWD	6.89E-08	-
GNPDA2	6.95E-08	+
ARHGEF2	7.048E-08	-
TMEM39B	7.116E-08	-
ARMCX6	7.16E-08	+
CYBRD1	7.168E-08	+
GLI4	7.236E-08	-
FAP	7.253E-08	+
TM9SF1	7.53E-08	+
CASP4	7.593E-08	+
TRIM2	7.609E-08	-
ANAPC10	7.689E-08	+
MORC3	7.706E-08	+
NR2C2	7.722E-08	-
SGPP1	7.974E-08	+
GPT2	8.015E-08	-
UPF2	8.019E-08	-
ZBTB38	8.063E-08	+
BAZ1B	8.096E-08	-
ING2	8.185E-08	+
VAX2	8.19E-08	-
MRPS27	8.263E-08	+
LMBRD2	8.333E-08	+
KANK4	8.355E-08	-
POLR1C	8.377E-08	-

TRAPPC4	8.559E-08	+
MRPL55	8.592E-08	-
HOOK1	8.703E-08	-
H2AFX	8.753E-08	-
FAM71E1	8.805E-08	-
NTRK3	8.859E-08	-
PRPS1	9.188E-08	+
SNX13	9.205E-08	+
DALRD3	9.216E-08	+
CPSF1	9.232E-08	-
B4GALNT4	9.311E-08	-
C6orf211	9.311E-08	+
CD93	9.525E-08	+
AGR3	9.621E-08	+
SLC25A11	9.681E-08	-
ACCN3	9.737E-08	-
CR614803	9.743E-08	+
ZNF358	9.817E-08	-
CCDC126	9.925E-08	+
SRM	9.961E-08	-
ENPP4	1E-07	+
RNF135	1.001E-07	+
EFNB1	1.002E-07	-
MBD3	1.007E-07	-
PPP1R14B	1.023E-07	-
ID3	1.026E-07	+
SRP14	1.026E-07	+
BRD3	1.036E-07	-
ELK4	1.04E-07	-
APPL2	1.04E-07	+
MCM7	1.051E-07	-
VGLL4	1.054E-07	-
MFGE8	1.068E-07	-
SHC4	1.086E-07	-
TLR3	1.112E-07	+
CCDC123	1.118E-07	-
ABCG1	1.119E-07	+
SCRIB	1.131E-07	-
CCDC53	1.134E-07	+
NOP14	1.147E-07	-
CHMP4B	1.15E-07	+
KLHL2	1.156E-07	+
TMBIM6	1.158E-07	+
NELF	1.167E-07	-
CLEC4A	1.168E-07	+
GALC	1.17E-07	+

BG564551	1.207E-07	-
HIC2	1.217E-07	-
CTSK	1.253E-07	+
CXorf40A	1.253E-07	+
PNPO	1.281E-07	+
PTCH1	1.292E-07	-
PLEKHG6	1.292E-07	-
ZNF410	1.293E-07	+
C16orf72	1.296E-07	+
ZNFPHEX133	1.302E-07	-
RBM15	1.308E-07	-
SLC29A3	1.309E-07	+
WDR26	1.323E-07	+
BRE	1.34E-07	+
ZFYVE16	1.349E-07	+
CCDC86	1.354E-07	-
CTSH	1.357E-07	+
ARID1A	1.357E-07	+
SLCO2B1	1.363E-07	+
TONSL	1.379E-07	-
ERCC8	1.392E-07	+
CERK	1.407E-07	-
TFF3	1.411E-07	+
CHST15	1.421E-07	+
AL133627	1.421E-07	-
TMSB4X	1.433E-07	+
DERL2	1.445E-07	+
POLQ	1.477E-07	-
DUS3L	1.491E-07	-
SLC4A3	1.494E-07	-
ZNF687	1.507E-07	-
PGK1	1.512E-07	+
RASA1	1.529E-07	+
HLA-DQA1	1.534E-07	+
SNORD56	1.54E-07	-
MFAP3	1.547E-07	+
UBE2G1	1.547E-07	+
DCK	1.548E-07	+
CPVL	1.576E-07	+
ICOSLG	1.579E-07	-
DDA1	1.589E-07	-
MUTYH	1.589E-07	-
CBFA2T2	1.603E-07	-
PARVB	1.607E-07	-
MAP2K1	1.609E-07	+
C13orf27	1.618E-07	+

CMBL	1.621E-07	+
HSPA8	1.658E-07	+
FLJ44124	1.658E-07	-
BQ709011	1.666E-07	-
PTBP1	1.677E-07	-
ARID4B	1.693E-07	-
RBM42	1.714E-07	-
TGFB3	1.722E-07	+
PPA2	1.728E-07	+
DCAF15	1.76E-07	-
RUNDC2A	1.771E-07	+
RDBP	1.784E-07	-
TICAM2	1.809E-07	+
SC4MOL	1.818E-07	+
PRPF19	1.881E-07	-
THAP7	1.885E-07	-
CPEB4	1.894E-07	+
NCK2	1.914E-07	-
DQ577523	1.94E-07	-
ART3	1.947E-07	-
DYNLT3	1.96E-07	+
LITAF	1.984E-07	+
ZEB2	1.988E-07	+
SON	1.995E-07	+
SPOPL	2.012E-07	+
KRT9	2.024E-07	-
SBK1	2.032E-07	-
PARP9	2.039E-07	+
HBXIP	2.052E-07	+
TTLL12	2.064E-07	-
THBS1	2.074E-07	+
ST3GAL4	2.081E-07	-
DNAJB12	2.092E-07	+
RPUSD3	2.103E-07	-
RAD9A	2.167E-07	-
PPP1R14C	2.169E-07	-
CD237904	2.173E-07	-
EPHB6	2.204E-07	-
RPS19	2.223E-07	-
C19orf47	2.234E-07	-
NOTCH1	2.251E-07	-
ACOX2	2.257E-07	+
CIC	2.295E-07	-
LOC202181	2.31E-07	-
SYNC	2.312E-07	+
DYNLL1	2.339E-07	+

TC2N	2.344E-07	+
CLIC1	2.359E-07	+
RNF165	2.364E-07	-
CHAF1A	2.395E-07	-
RNF150	2.403E-07	-
LSM3	2.446E-07	+
NMT2	2.477E-07	-
ALDOB	2.479E-07	-
FAM127B	2.481E-07	-
DUSP1	2.507E-07	+
C1orf46	2.52E-07	-
AURKB	2.524E-07	-
COMMD8	2.544E-07	+
IL34	2.555E-07	-
FUCA1	2.572E-07	+
PLXNB1	2.578E-07	-
TMEM167B	2.597E-07	+
PRPF38A	2.61E-07	-
ANKRD23	2.614E-07	-
SERBP1	2.615E-07	-
PPM1G	2.641E-07	-
MAT2B	2.684E-07	+
TMEM55B	2.692E-07	+
SMCR7L	2.692E-07	-
RALBP1	2.703E-07	-
BZW1	2.719E-07	+
RPRML	2.722E-07	-
CA12	2.735E-07	+
RAD23A	2.764E-07	-
CTU1	2.787E-07	-
RCBTB2	2.796E-07	+
ZNF292	2.812E-07	-
AW020614	2.835E-07	-
POU5F1	2.841E-07	-
MORF4L1	2.849E-07	+
INCENP	2.856E-07	-
PHGDH	2.879E-07	-
AX747098	2.887E-07	-
XYLB	2.888E-07	-
TBC1D10B	2.89E-07	-
NDUFV1	2.9E-07	-
PRKAG1	2.922E-07	+
FLJ41455	2.923E-07	-
FUT8	2.926E-07	+
TM2D1	2.928E-07	+
SHANK3	2.931E-07	-

CTPS	2.966E-07	-
SRGAP2	2.966E-07	-
PLEKHA3	2.979E-07	+
SLC25A46	2.99E-07	+
TMEM158	2.995E-07	-
KHDRBS1	3.008E-07	+
ERLEC1	3.024E-07	+
C1orf135	3.037E-07	-
POC1B	3.052E-07	+
HINT1	3.056E-07	+
CEBPG	3.068E-07	-
IKZF5	3.082E-07	+
ARHGAP12	3.096E-07	+
FBXO10	3.108E-07	-
TMEM126B	3.127E-07	+
SKI	3.161E-07	-
TRIT1	3.162E-07	-
PLAC8L1	3.177E-07	-
ATG3	3.2E-07	+
ATXN2L	3.221E-07	-
LRP3	3.231E-07	-
ATP6AP2	3.251E-07	+
NRP1	3.34E-07	+
MRPS34	3.35E-07	+
SLFN12	3.397E-07	+
SLC25A24	3.429E-07	+
CARM1	3.446E-07	-
TOB2	3.464E-07	-
LAMTOR1	3.535E-07	+
MRPS2	3.569E-07	-
WDR46	3.571E-07	-
CHP	3.574E-07	+
NES	3.586E-07	-
GPR68	3.587E-07	+
KIAA0247	3.59E-07	+
UBXN8	3.594E-07	+
TDG	3.596E-07	-
PCNP	3.617E-07	+
TNFAIP8	3.643E-07	+
FYCO1	3.653E-07	+
NR3C1	3.682E-07	+
AP1G1	3.725E-07	+
QSOX2	3.753E-07	-
ANO6	3.771E-07	+
PPAP2B	3.783E-07	+
SLC7A8	3.786E-07	+

ATP6V0C	3.789E-07	+
TJP1	3.794E-07	+
ALKBH7	3.802E-07	+
DUSP8	3.816E-07	-
BC071719	3.828E-07	+
MGAT3	3.835E-07	-
FAM100B	3.843E-07	-
C11orf68	3.852E-07	-
SLX1A	3.858E-07	+
SEPHS2	3.867E-07	+
MYPOP	3.881E-07	-
OSTCP1	3.885E-07	-
CCNH	3.906E-07	+
MITD1	3.914E-07	+
SLC39A6	3.952E-07	+
