# Supplementary material for: Trimodal distribution of arylamine N-acetyltransferase 1 mRNA in breast cancer tumors: association with overall survival and drug resistance
Source: BMC Genomics. 2018 Jul 3;19:513. doi: 10.1186/s12864-018-4894-4 (PMC6029418; doi:10.1186/s12864-018-4894-4)
Supplement: Supplementary file 5 — Table S2. The Cox proportional hazard regression models based on survival for NAT1 sub-populations, tumour characteristics and treatment. (PDF 121 kb) [file 12864_2018_4894_MOESM5_ESM.pdf]

Table S2. The Cox proportional hazard regression models based on survival for NAT1 sub-populations, tumour characteristics and treatment.

Variable	Univariate		Multivariate	
	HR (95% CI)	p	HR (95% CI)	p
NAT1	0.75 (0.67-0.84)	<0.0001	0.84 (0.74-0.95)	0.007
Tumor size	1.02 (1.01-1.02)	<0.0001	1.01 (1.007-1.017)	<0.0001
Age at Diagnosis	1.00 (0.99-1.01)	0.85	1.03 (1.02-1.04)	<0.0001
Menopause	0.88 (0.71-1.24)	0.26	0.71 (0.51-1.00)	0.054
Chemotherapy (No vs Yes)	0.41 (0.33-0.50)	<0.0001	0.62 (0.54-0.70)	<0.0001
Radiotherapy (No vs Yes)	0.84 (0.69-1.02)	0.085	1.04 (0.84-1.28)	0.88
Hormone Therapy (No vs Yes)	0.98 (0.89-1.09)	0.75	0.93 (0.83-1.04)	0.19

HR = hazard ratio; CI = confidence intervals
